# Microgel reinforced zwitterionic hydrogel coating for blood-contacting biomedical devices

**DOI:** 10.1038/s41467-022-33081-7

**Published:** 2022-09-12

**Authors:** Mengmeng Yao, Zhijian Wei, Junjin Li, Zhicheng Guo, Zhuojun Yan, Xia Sun, Qingyu Yu, Xiaojun Wu, Chaojie Yu, Fanglian Yao, Shiqing Feng, Hong Zhang, Junjie Li

**Affiliations:** 1 School of Chemical Engineering and Technology, Tianjin University, 300350 Tianjin, China; 2 Frontiers Science Center for Synthetic Biology and Key Laboratory of Systems Bioengineering (Ministry of Education), Tianjin University, 300350 Tianjin, China; 3 International Science and Technology Cooperation Base of Spinal Cord Injury, Department of Orthopedic Surgery, Tianjin Medical University General Hospital, Tianjin, China; 4 Department of Orthopaedics, Qilu Hospital, Cheeloo College of Medicine, Shandong University, 250012 Jinan, Shandong China; 5 Shandong University Centre for Orthopaedics, Cheeloo College of Medicine, Shandong University, 250012 Jinan, Shandong China; 6 School of Materials Science and Engineering, East China Jiaotong University, 330013 Nanchang, China

**Keywords:** Biomedical materials, Implants, Biomaterials

## Abstract

Zwitterionic hydrogels exhibit eminent nonfouling and hemocompatibility. Several key challenges hinder their application as coating materials for blood-contacting biomedical devices, including weak mechanical strength and low adhesion to the substrate. Here, we report a poly(carboxybetaine) microgel reinforced poly(sulfobetaine) (pCBM/pSB) pure zwitterionic hydrogel with excellent mechanical robustness and anti-swelling properties. The pCBM/pSB hydrogel coating was bonded to the PVC substrate via the entanglement network between the pSB and PVC chain. Moreover, the pCBM/pSB hydrogel coating can maintain favorable stability even after 21 d PBS shearing, 0.5 h strong water flushing, 1000 underwater bends, and 100 sandpaper abrasions. Notably, the pCBM/pSB hydrogel coated PVC tubing can not only mitigate the foreign body response but also prevent thrombus formation ex vivo in rats and rabbits blood circulation without anticoagulants. This work provides new insights to guide the design of pure zwitterionic hydrogel coatings for biomedical devices.

## Abstract (web summary)

Zwitterionic hydrogels are nonfouling and hemocompatibility but several key challenges such as weak mechanical strength and low adhesion hamper their application as coating materials for devices. Here, the authors report a microgel reinforced zwitterionic hydrogel with excellent mechanical robustness and anti-swelling properties.

## Introduction

Blood-contacting biomedical devices (e.g., interventional catheters, vascular stents, and mechanical heart valves) have been widely used in hemodialysis^1^, drug delivery^2^, extracorporeal circulation^3^, and other clinical treatments^4^. However, thrombosis and infection are still the main challenges of blood-contacting biomedical devices in clinical applications^5^. In the past few decades, blood-contacting biomedical devices coated with anticoagulants and antibiotics have proven to mitigate thrombosis and infection^6^. Unfortunately, excessive anticoagulants and antibacterial agents, particularly when administered systemically over long term, may cause antibiotic resistance, postoperative bleeding, and thrombocytopenia^7^. Therefore, there exists an urgent clinical need for a method that replaces anticoagulants and antibiotics.

The adhesion of proteins, bacteria, and platelets on blood-contacting biomedical devices is the main reason for thrombosis and infection^8^. Many studies have shown that zwitterionic polymers are ideal candidates to prevent the formation of thrombosis and the occurrence of infection, owing to their superhydrophilic properties via equal amounts of positive and negative charged moieties on the same side chain^9–11^. Generally, the formation of zwitterionic polymer brushes on blood-contacting biomedical devices is a promising strategy to resist biofouling. Zhang et al.^12^ prepared a poly(sulfobetaine methacrylate) brush on gold surface as antifouling coatings through atom transfer radical polymerization. Similarly, Huang et al.^13^ prepared ultra-low fouling poly(carboxybetaine) brushes for sensing platforms via surface-initiated atom transfer radical polymerization. However, the thickness of these brushes usually does not exceed 100 nm^14^. In addition, the complex preparation processes (e.g., strict oxygen removal or the use of metal catalysts) limit its further application^15^. Compared with polymer brush, hydrogel coatings show obvious advantages, for example, their thickness, hydrophilicity, mechanical properties, and lubricity can be reasonably regulated^16–18^. Nevertheless, the application of zwitterionic hydrogel coatings in anti-thrombosis and anti-infection medical devices is still limited. As a hydrogel coating, it is essential that zwitterionic hydrogels have robust mechanical properties and strong adhesion to blood-contacting biomedical devices^19^. In fact, the mechanical properties of zwitterionic hydrogels are quite weak due to their superhydrophilic characteristics^20,21^. Some non-zwitterionic components are usually introduced to improve mechanical properties. Haraguchi et al.^22^ showed that the tensile strength of sulfobetaine polyacrylamide/inorganic clay nanocomposite hydrogels improved to 71.3 kPa from 19.0 kPa. Unfortunately, this method often sacrifices the antifouling properties of zwitterionic hydrogels^23^. Hence, their long-term mechanical stability and excellent antifouling properties are not combined but compromised with each other. On the other hand, zwitterionic hydrogels exhibit a high swelling ratio due to their superhydrophilic properties, which is often inconsistent with the swelling behavior of the substrate and further causes the hydrogel coating to fall off the substrate^24^. Therefore, maintaining the anti-swelling state of the zwitterionic hydrogel in the blood environment is also a key requirement to improve the stability of the hydrogel coating. To combine the high mechanical strength and anti-swelling properties, Jiang et al.^25^ developed pure zwitterionic hydrogels through “swelling” and “locking” mechanisms, which had excellent mechanical properties and anti-swelling performance as bulk hydrogels. However, they were unfavorable for the construction of the coating due to its unique multistep curing process. Compared with bulk hydrogels, microgels with a high specific surface area were an ideal candidate to improve the mechanical strength of hydrogels^26^. Gong et al.^27,28^ found that microgel-reinforced hydrogels can effectively relieve stress concentrations. This provides the possibility to prepare microgel-based pure zwitterionic hydrogel coatings.

Strong adhesion of hydrogel coatings is a critical requirement for blood-contacting biomedical devices^29^. The surface of most blood-contacting biomedical devices (e.g., PVC, PET, and PU) is hydrophobic and does not contain reactive groups^30^. Therefore, the adhesion strength of the hydrogel coating through physical interaction with the substrate is rather weak^31^. Generally, bridging molecules (such as silane coupling agents and dopamine) can improve the adhesion of hydrogels to substrates through covalent bonding^32^. In our previous study, the starch-based zwitterionic hydrogel coating was covalently anchored through the oxidative polymerization of dopamine^33^. However, the bridging molecule layer between hydrogels and substrates is always affected by the complex microenvironment (e.g., pH and solubility), which is unfavorable for long-term application. Initiating polymerization from the substrate network can reduce the interface layer and improve the bonding strength of the coating. For example, Zhao et al.^34,35^ developed a hydrogel coating preparation technology via the surface initiation method to interpenetrate the substrate polymer and the hydrogel coating network at the interface, which provided a feasible approach to improve the interface bonding strength of hydrogel coatings.

Herein, we are committed to developing a microgel-reinforced zwitterionic hydrogel coating for blood-contacting devices. The poly(carboxybetaine) microgel (pCBM) was first prepared by inverse miniemulsion polymerization, and then combined with poly(sulfobetaine) (pSB) to construct pure zwitterionic hydrogels (pCBM/pSB) with mechanical robustness and anti-swelling properties. In this system, the pSB as a continuous phase passes through the pCBM (acted as a cross-linking point) to reinforce the mechanical strength. Based on these results, the pCBM/pSB hydrogel was coated on the polyvinyl chloride (PVC) substrate via the simultaneous polymerization of [2-(methacryloyloxy) ethyl] dimethyl-(3-sulfopropyl) ammonium hydroxide (SBMA) monomer in both the pCBM/pSB pre-gel solution and swelling PVC interface layer. The entanglement network and grafting reaction between the pSB and PVC chain would significantly improve the bonding strength of the substrate and coating. The formation mechanism, surface properties, mechanical strength, and stability of the obtained pCBM/pSB hydrogel coating were investigated in detail. Finally, the ex vivo antithrombogenicity behaviors of the prepared pCBM/pSB hydrogel coating were investigated using the extracorporeal circuit (ECC) model of SD rats and New Zealand white rabbits.

## Results

### Formation and properties of pCBM/pSB zwitterionic hydrogel

**Fig. 1:**
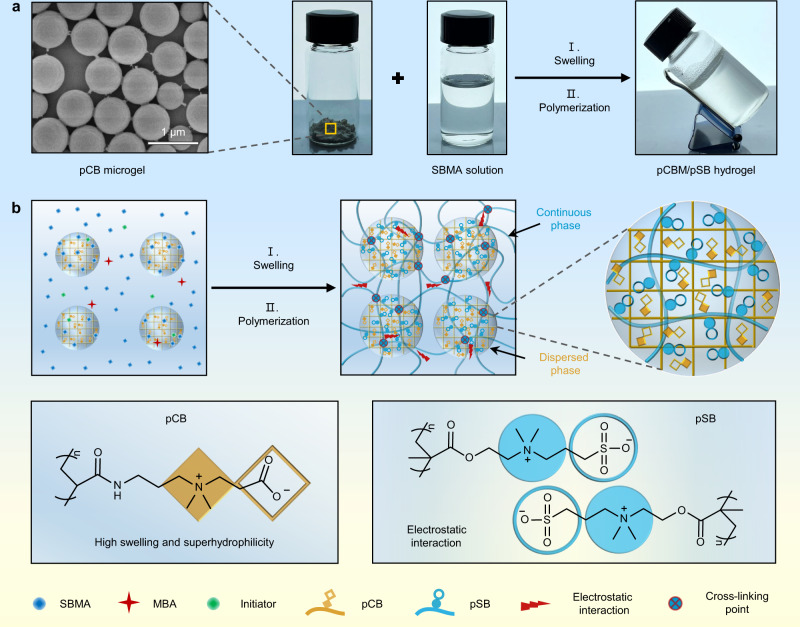
Design principle of mechanically robust pure zwitterionic pCBM/pSB hydrogel. **a** Photograph of the preparation of the pCBM/pSB hydrogel. **b** Network structure and the preparation process of the pCBM/pSB hydrogel.

Due to their superhydrophilic nature, zwitterionic hydrogels generally have high swelling behavior and weak mechanical strength. To overcome these shortcomings, we reported a zwitterionic microgel enhanced pure zwitterionic hydrogel with mechanical robustness and anti-swelling properties. The pCBM was first prepared by inverse miniemulsion polymerization, and then dry pCBM with 641 ± 65 nm in diameter (Fig. 1a and Supplementary Fig. 1a) was dispersed and swelled in SBMA monomer solution to obtain paste-like pre-gel solution. Owing to the high specific surface area and superhydrophilic properties, SBMA monomer could quickly enter pCBM. In contrast, dry SB microgels tend to agglomerate in the SB pre-gel solution due to the polyelectrolyte effect (Supplementary Fig. 1b). After polymerization of SBMA, the pSB chain passed through the pCBM and formed a uniform network structure, resulting in the obtained pCBM/pSB hydrogel had a two-phase structure (Fig. 1b). As shown in Supplementary Fig. 2, many bumped microstructures could be observed on the cross-section of pCBM/pSB hydrogel due to the introduction of pCBM. The sparsely cross-linked pSB network acted as a continuous phase, which restricted deformation through “locking” the entire hydrogel by the strong inter- and intramolecular electrostatic interactions. The dispersed phase was a double-network microgel composed of interpenetrating pSB and pCB chains. Different from the traditional nano-particles enhancement mechanism, the hydrophilicity of pCBM contributed to its uniform dispersion in the composite hydrogel network. In addition, the microgel phase could not only act as the cross-linking sites to improve the mechanical strength, but also dissipate energy through the destruction and reconstruction of the electrostatic interaction between pCB and pSB, which further avoided the stress concentration and improved the mechanical properties.

**Fig. 2:**
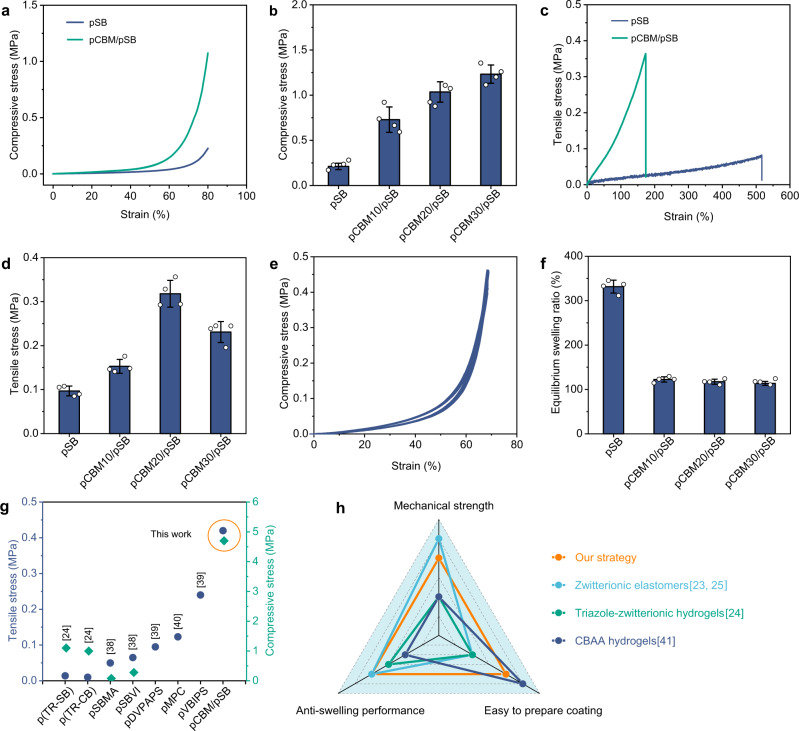
Mechanical and anti-swelling properties of the pCBM/pSB hydrogels. **a** Compressive and (**c**) tensile stress–strain curves of the pCBM/pSB hydrogel and the pSB hydrogel. **b** Compressive stress (*n* = 4) and (**d**) tensile stress (*n* = 4) of the pCBM/pSB hydrogel with varying pCBM concentrations. **e** Representative loading–unloading compression curves (100 runs) of the pCBM20/pSB hydrogel. **f** Equilibrium swelling ratios of the pCBM/pSB hydrogels with varying pCBM concentrations (*n* = 4). **g** Comparison diagram of the compressive stress at 80% strain and tensile stress at fracture of other pure zwitterionic hydrogels^24,38–40^. **h** Comparison of the preparation of other pure zwitterionic hydrogels between this work and other strategies^23–25,41^, and in terms of their mechanical strength, anti-swelling, and easy-to-prepare coating properties. Data presented as mean ± SD in (**b**, **d**, **f**). Source data are provided as a Source Data file.

The cross-linking degree of pCBM is a critical factor parameter to modulate the mechanical properties of pCBM/pSB hydrogels. When the concentration of MBA was 1 mol% (relative to pCBM), the compressive stress of pCBM/pSB hydrogel was 0.59 ± 0.02 MPa, and it increased to 1.68 ± 0.15 MPa when the MBA concentration increased to 4 mol% (Supplementary Fig. 3a, b). However, with a further increase of MBA concentration (7 mol%), the compressive stress slightly reduced to 1.46 ± 0.13 MPa and fractured at 80.6% strain. This was because the pCBM with high MBA concentration caused the inability of the pSB network to transmit enough force, which resulted in the pCBM breaking first. In this case, pCBM could not act as a sacrificial bond. Therefore, MBA with a concentration of 4 mol% was selected to prepare pCBM/pSB hydrogels and coatings. Moreover, the pCBM concentrations is also the main parameter to modulate the mechanical properties of pCBM/pSB hydrogels. The compressive strength of the pCBM/pSB hydrogel increased with the increase of the pCBM concentration. The compressive strength of pCBM10/pSB hydrogel improved to 0.73 ± 0.15 MPa compared with the pSB hydrogel (0.23 ± 0.05 MPa). As expected, the compressive strength of pCBM20/pSB and pCBM30/pSB hydrogel was significantly improved to 1.03 ± 0.11 MPa and 1.23 ± 0.10 MPa, respectively. Interestingly, pCBM20/pSB hydrogel could be smoothly compressed to a strain of 95% for 1 h and restored to its original shape after relaxation (Supplementary Fig. 4a). However, pCBM30/pSB hydrogel was easily broken by compressing to 93.3 ± 0.3% strain (Supplementary Fig. 4b). This might be due to stress concentration caused by the higher pCBM concentration. Similarly, the tensile curve of the pCBM20/pSB hydrogel behaves in an elastic manner without obvious stress yielding (Fig. 2c). Compared with the pSB hydrogel, the fracture strength of the pCBM20/pSB hydrogel increased to 0.32 ± 0.03 MPa from 0.09 ± 0.01 MPa (Fig. 2d), while the fracture strain reduced to 225 ± 45% (Supplementary Fig. 4c). The tensile strength of the pCBM30/pSB hydrogel (0.23 ± 0.02 MPa) was lower than that of pCBM20/pSB hydrogel (0.32 ± 0.03 MPa). Furthermore, the pCBM20/pSB hydrogels also exhibited the highest compressive and tensile modulus (Supplementary Fig. 4d, e). Notably, the compression curves (70% strain) of pCBM20/pSB overlapped in at least 100 cycles (Fig. 2e), indicating effective recovery after loading and no permanent deformation.

The swelling resistance of zwitterionic hydrogels was rather weak because of their superhydrophilic characteristics. As shown in Fig. 2f, the swelling ratio of the pSB hydrogel was ~333%. As expected, the pCBM/pSB hydrogel showed a low swelling ratio with the introduction of pCBM. The SEM results further revealed that the pore size of the pCBM/pSB hydrogel was much smaller than that of the pSB hydrogel after swelling, suggesting that the pCBM/pSB hydrogel network was denser than the pSB hydrogel network (Supplementary Fig. 5). This phenomenon was mainly caused by the incorporation of pCBM, which restricted the movement of the pSB chains, resulting in a lower swelling ratio.

Owing to the special network structure enhanced by pCBM, the prepared pCBM/pSB hydrogels outperformed most existing zwitterionic hydrogels, especially in terms of maximum compressive stress and tensile stress (Fig. 2g). In addition, compared with previously reported pure zwitterionic materials (Fig. 2h), the straightforward and controllable zwitterionic hydrogel preparation strategy in this study was superior in terms of mechanical strength, swelling resistance, and easy-to-prepare hydrogel coating’s overall properties. More importantly, the pCBM/pSB pre-gel solution was easy to store, transport, and coat on the substrate, which was ideal for application in the surface engineering of blood-contacting biomedical devices.

### Formation of pCBM/pSB hydrogel coatings

**Fig. 3:**
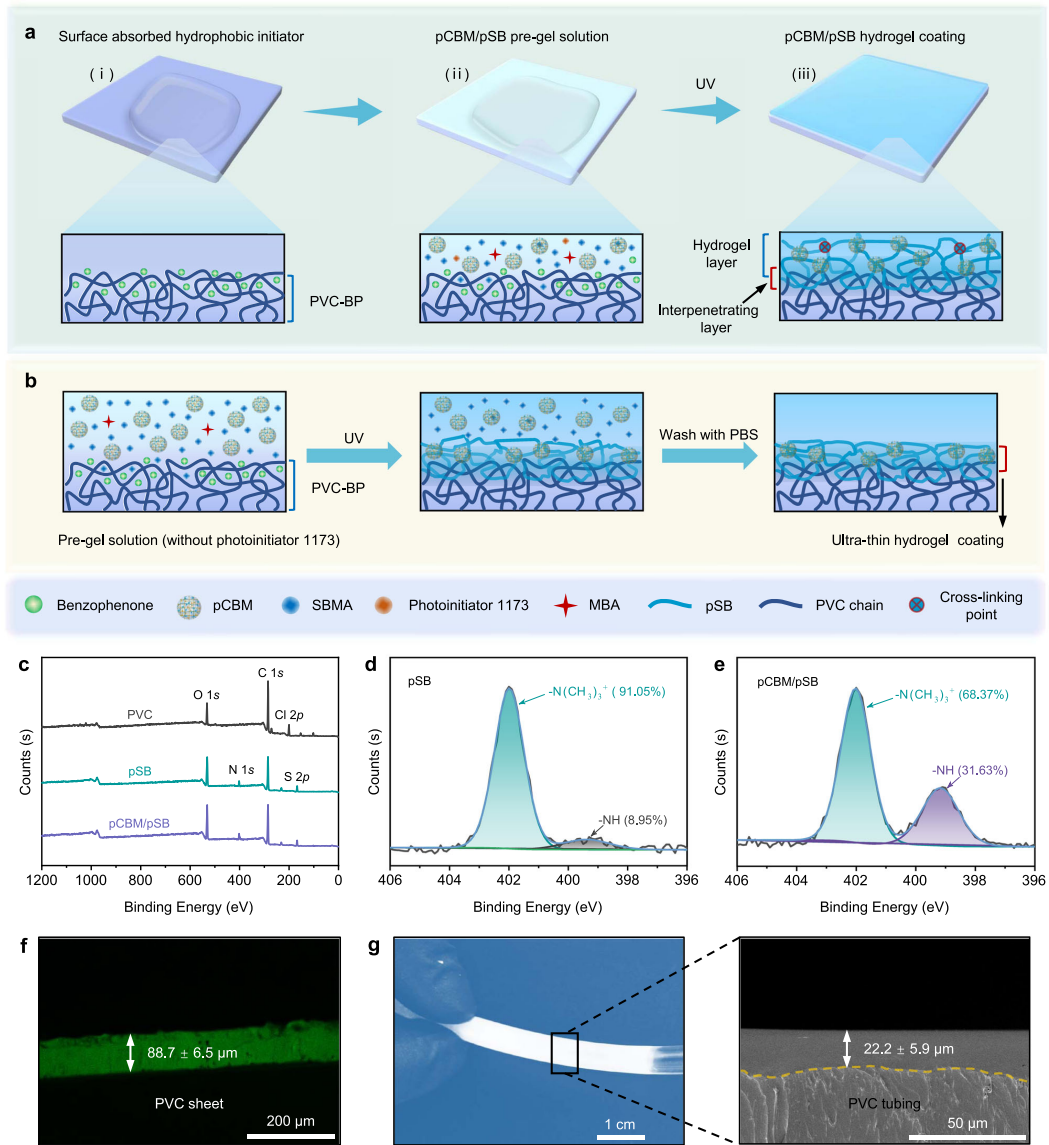
Formation of pCBM/pSB hydrogel coatings. **a** Schematic illustration of the pCBM/pSB hydrogel coating preparation procedures. (i) The PVC substrates were treated with acetone containing 5 wt% hydrophobic initiators (benzophenone). (ii) PVC substrates were covered with pCBM/pSB pre-gel solution containing hydrophilic initiators (photoinitiator 1173). (iii) After UV-initiated polymerization, the pSB/PVC interpenetrating layer and pCBM/pSB hydrogel coating were formed on the PVC substrates. **b** Preparation process of ultrathin pCBM/pSB hydrogel coating. **c** X-ray photoelectron spectroscopy survey scan of PVC substrate, pSB hydrogel coating, and pCBM/pSB hydrogel coating. High-resolution spectra of N 1 *s* of (**d**) pSB and (**e**) pCBM/pSB hydrogel coating. **f** Fluorescence image of the cross-section of pCBM/pSB hydrogel coatings on PVC sheet by using a 0.1-mm thick pre-gel solution. Measurements in (**f**) were repeated three times independently with similar results. **g** Digital photo and SEM image of the cross-section of pCBM/pSB hydrogel coatings on PVC tubing. Measurements in (**g**) (right panel) were repeated three times independently with similar results. Source data are provided as a Source Data file.

A key challenge in the application of pCBM/pSB hydrogel coatings is to achieve strong adhesion to blood-contacting biomedical devices. Here, we adopted a straightforward surface initiation strategy to improve the bonding strength between the pCBM/pSB hydrogel and the PVC substrate. As shown in Fig. 3a, the PVC substrate was first pretreated with a hydrophobic photoinitiator benzophenone (BP) solution. The BP could penetrate into the swollen PVC substrate and form a diffusion layer. When the pCBM/pSB pre-gel solution was coated on the surface of the PVC-BP substrate, the SBMA monomer then diffused into the swollen PVC interface layer. Using hydrophobic BP and hydrophilic photoinitiator 1173 as initiators, the SBMA monomers in both pre-gel solution and on the PVC-BP interface layer were simultaneously polymerized to construct the pSB network in the presence of the MBA crosslinker. Therefore, the pCBM/pSB hydrogel coating was formed on PVC substrate by the entanglement network between the pSB and PVC chains. Moreover, the hydrophobic photoinitiator BP served as a triplet biradical could induce pSB grafted from the PVC chain via the C–H insertion grafting reaction mechanism^34,35^. When the hydrogel coating was wrecking, the strong interface enabled energy transfer from the surface to the bulk pCBM/pSB hydrogel, which resisted detachment through internal energy dissipation mechanisms. For the pCBM/pSB pre-gel solution without a hydrophilic photoinitiator 1173 (Fig. 3b), the excess reaction solution could be removed by washing with PBS after polymerization to obtain a uniform and ultrathin pCBM/pSB hydrogel coating. By this method, we could easily prepare a controllable thickness, mechanically robust, and strong adhesion pCBM/pSB hydrogel coating.

To verify the formation of pCBM/pSB hydrogel coatings, the surface chemical compositions were studied using ATR-FTIR and XPS. Compared with PVC, the asymmetric vibration peak of S = O at 1033 cm^−1^ appeared in both the pSB and pCBM/pSB hydrogel coatings (Supplementary Fig. 6). After the hydrogel was coated on PVC, the Cl 2*p* signal of the PVC substrate completely disappeared (Fig. 3c), while N 1 *s* and S 2*p* appeared. The N 1 *s* were fitted into two peaks: –N(CH_3_)_3_^+^ (402.2 eV) and –NH (399.9 eV), while the –NH group corresponded to the nitrogen atom on the pCBM moieties. The percentage nitrogen of the –NH group in pCBM/pSB hydrogel coatings improved to 31.63% from 8.59% of the pSB hydrogel coatings (Fig. 3d, e), indicating that pCBM was introduced into the hydrogel coating. In addition, the thickness of the pCBM/pSB hydrogel coating could be obviously observed via the fluorescence and SEM image of the cross-section (Fig. 3f and Supplementary Fig. 7). Notably, the pCBM/pSB hydrogel could effectively and easily be coated on the inner wall of commercial blood circulation PVC tubing (50 cm length), and the thickness of pCBM/pSB hydrogel coating was 22.2 ± 5.9 μm (Fig. 3g).

### Surface properties of pCBM/pSB hydrogel coatings

**Fig. 4:**
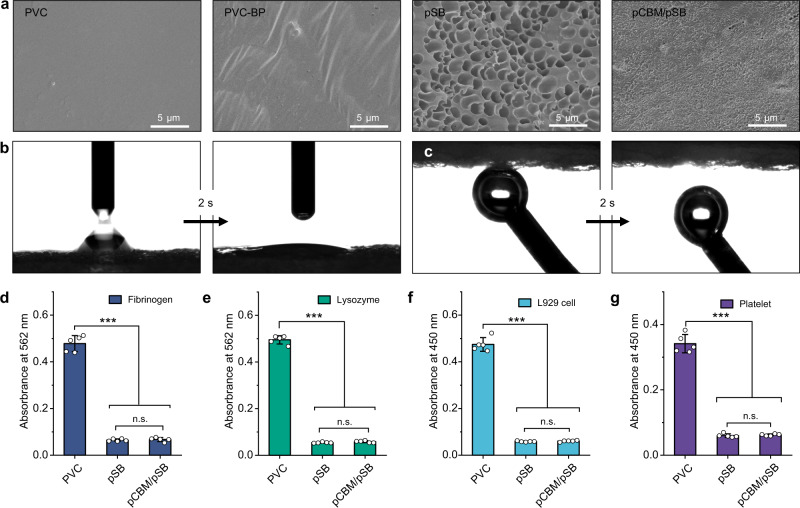
Surface properties of the pCBM/pSB hydrogel coatings. **a** SEM images of PVC, PVC-BP, pSB hydrogel coating, and pCBM/pSB hydrogel coating. Measurements in (**a**) were repeated three times independently with similar results. (**b**) Hydrophilicity and (**c**) underwater superaerophobicity of the pCBM/pSB hydrogel coatings. The relative adhesion amount of (**d**) fibrinogen (*n* = 5), (**e**) lysozyme (*n* = 5), (**f**) L929 cells (*n* = 5), and (**g**) platelets (*n* = 5) of PVC substrate, pSB hydrogel coating, and pCBM/pSB hydrogel coating. Data presented as mean ± SD and analyzed using a one-way ANOVA with Tukey’s post hoc test in (**d**, **e**, **f**, **g**), ***P < 0.001, n.s.: no significant difference at *P* > 0.05. **d** *P* = 0.6198 (pSB vs pCBM/pSB, fibrinogen), *P* < 0.001 (PVC vs pCBM/*p*SB, PVC vs pSB, fibrinogen). **e** *P* = 0.1116 (pSB vs pCBM/pSB, lysozyme), *P* < 0.001 (PVC vs pCBM/pSB, PVC vs pSB, lysozyme). **f** *P* = 0.3095 (pSB vs pCBM/pSB, L929 cells), *P* < 0.001 (PVC vs pCBM/pSB, PVC vs pSB, L929 cells). **g** *P* = 0.4564 (pSB vs pCBM/pSB, platelets), *P* < 0.001 (PVC vs pCBM/pSB, PVC vs pSB, platelets). Source data are provided as a Source Data file.

The pristine PVC surface was smooth and flat (Fig. 4a), and it became rough after being treated by acetone solution with benzophenone. The pSB hydrogel coating showed a uniform multi-porous structure. In contrast, the pCBM/pSB hydrogel coating became dense and non-porous. The pCBM/pSB hydrogel coating significantly increased the hydrophilicity of PVC. The water contact angle decreased from 97.3 ± 2.5° of the pristine PVC to 8.1 ± 3.3° of the pCBM/pSB hydrogel coating and rapidly dropped to 0 within 2 s (Fig. 4b and Supplementary Fig. 8). Moreover, the underwater gas contact angle of the pCBM/pSB hydrogel coating remained at 160.3 ± 2.1° and spontaneously bounced off within 2 s of contact with the bubble (Fig. 4c). These results indicated that the pCBM/pSB hydrogel coating exhibited hydrophilic properties.

Owing to the superhydrophilic properties, the fibrinogen and lysozyme adsorption of the pCBM/pSB hydrogel coating significantly reduced by 86.3% and 90.7% (*P* < 0.001), showing similar nonfouling abilities to the pSB hydrogel coating (Fig. 4d, e). In addition, few L929 cells, platelets, and bacterial adhesions were observed on both the pSB and pCBM/pSB hydrogel coating surfaces, while they adhered in a large quantity on the surfaces of the pristine PVC (Fig. 4f, g and Supplementary Fig. 9–11). These results showed that the pCBM/pSB hydrogel coating exhibited excellent nonfouling properties.

### Stability of pCBM/pSB hydrogel coatings

**Fig. 5:**
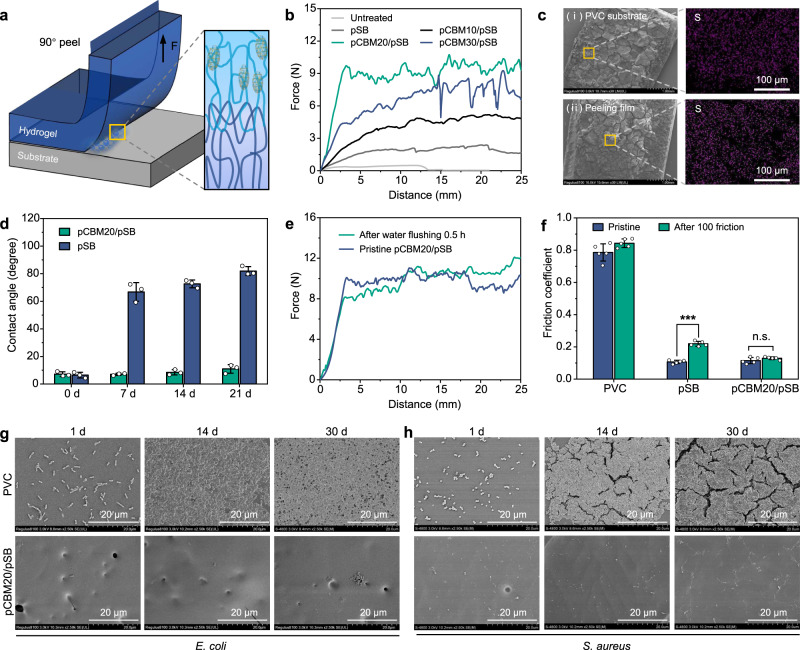
Mechanical stability and long-term antifouling properties of the pCBM/pSB hydrogel coatings. **a** Schematic illustration of the peeling test on pCBM/pSB hydrogel coatings. **b** Force-displacement curves of the peeling tests of untreated PVC substrates, pSB, pCBM10/pSB, pCBM20/pSB, and pCBM30/pSB hydrogel coatings. **c** SEM image and S element mapping of (i) the side of the PVC substrate and (ii) the peeling film after undergoing the peel test. Measurements in (**c**) were repeated three times independently with similar results. **d** Water contact angle of the pSB and pCBM20/pSB hydrogel coatings after shearing for 7 d, 14 d, and 21 d (*n* = 3). **e** The force-displacement curves of the pCBM20/pSB hydrogel coating before and after 0.5 h of strong water flushing. **f** The friction coefficients of the pSB and pCBM20/pSB hydrogel coatings before and after 100 sandpaper abrasion tests (*n* = 5). Representative SEM images for biofilm formation of (**g**) *Escherichia coli* (*E. coli*) and (**h**) *Staphylococcus aureus* (*S. aureus*) adhesion on PVC and pCBM20/pSB hydrogel coatings after 1 d, 14 d, and 30 d of coculture. Measurements in (**g**, **h**) were repeated three times independently with similar results. Data presented as mean ± SD in (**d**, **f**) and analyzed using a one-way ANOVA with Tukey’s post hoc test in **f**, ***P < 0.001, n.s.: no significant difference at *P* > 0.05. **f** *P* = 0.1571 (pristine vs after 100 friction, pCBM20/pSB), *P* < 0.001 (pristine vs. after 100 friction, pSB). Source data are provided as a Source Data file.

The stability of pCBM/pSB hydrogel coatings is the main requirement for the successful applications of blood-contacting biomedical devices. Generally, the stability of the hydrogel coating mainly depends on the mechanical strength of the hydrogel and bonding strength with the substrate. In this study, the introduction of pCBM obviously improved the mechanical strength of the pSB hydrogel (Fig. 2). Moreover, the pCBM/pSB hydrogel coating was tightly combined with the PVC substrate through chain winding among PVC and pSB chains (Fig. 5a). The results suggested that the introduction of pCBM improved the bonding strength compared with the pSB hydrogel coating. In addition, the binding strength depended on the pCBM concentrations, and the pCBM20/pSB hydrogel coating showed a higher adhesion strength (Fig. 5b and Supplementary Fig. 12). In contrast, a higher pCBM concentration might lead to poor hydrogel network integrity and further reduce the adhesion energy. Notably, SEM results showed that pCBM20/pSB hydrogels appeared both on the PVC substrate surface and the peeling film after the peel test, indicating that the cracks mainly propagated through the pCBM20/pSB hydrogel (Fig. 5c and Supplementary Fig. 13a, b). Due to the entanglement between the pSB chain and the PVC chain, the interface bonding strength was much higher than that of the pCBM/pSB hydrogel.

In addition, the stability of pCBM20/pSB hydrogel coatings was evaluated under various conditions. After soaking in PBS for 24 h, the thickness of pCBM20/pSB hydrogel coating was 28.5 ± 4.7 μm, which did not show statistical differences compared with before swelling (22.2 ± 5.9 μm) (Supplementary Fig. 14). These results were consistent with the results of swelling experiments of pCBM20/pSB hydrogel, indicating that the pCBM20/pSB hydrogel coatings had good stability in an aqueous environment. After 7 d of shearing in PBS, the water contact angle of the pSB hydrogel coating increased to 66.7 ± 6.7°, suggesting that its initial superhydrophilic properties failed (Fig. 5d). Furthermore, its antibacterial adhesion ability also decreased significantly (Supplementary Fig. 15a–c). These results suggested that the pSB hydrogel coating had been destroyed in the water shearing environment. As expected, the pCBM20/pSB hydrogel coating was firmly fixed on the PVC substrate due to strong adhesion. Moreover, the pCBM20/pSB hydrogel coating still maintained the superhydrophilic properties and excellent antibacterial adhesion behaviors within 21 d water shear environment. In addition, the pCBM20/pSB hydrogel coating could withstand mechanical damage. The thickness and morphology of the pCBM20/pSB hydrogel coating had few changes after being bent 1000 times underwater (Supplementary Fig. 16a–c). Furthermore, the pCBM20/pSB hydrogel coating could also maintain good adhesion to the substrate even after 30 min of strong water flushing (Fig. 5e). Notably, the friction coefficient of the pSB hydrogel coating was increased to 0.22 (*P* < 0.05, Fig. 5f) after 100 sandpaper abrasion tests. In contrast, the pCBM20/pSB hydrogel coating was still maintained at lower friction coefficient values, which was attributed to its excellent stability and robust mechanical properties.

Microorganisms attach to the surface of biomedical equipment and can further cause many symptoms such as biofilm formation and infection. In this study, *S. aureus* and *E. coli* were used to evaluate the long-term anti-biofilm formation properties of the pCBM20/pSB hydrogel coating. The results showed that the PVC substrate was completely covered by the biofilm after 1 week (Fig. 5g, h). Intriguingly, in the 2-week and 1-month studies, not only no biofilm was formed on the surface of all pCBM20/pSB hydrogel coatings, but also almost no bacteria attached (Fig. 5g, h).

### Biocompatibility of pCBM/pSB hydrogel coatings

The biocompatibility of the pCBM/pSB hydrogel coating is an important parameter for blood-contacting devices., the viability in the pCBM/pSB hydrogel coatings group was always more than 90%, demonstrating its negligible cytotoxicity (Supplementary Fig. 17a). Moreover, the hemolysis rate of pCBM/pSB was <2% (Supplementary Fig. 17b). PVC tubing with and without pCBM/pSB hydrogel coating was subcutaneously implanted in SD rats for 1 month to evaluate its anti-inflammatory effects. The PVC substrate was wrapped by a layer of dense collagen capsule (yellow arrow, Supplementary Fig. 18a, b). The pCBM/pSB hydrogel coatings had a particularly low density of collagen deposition. Moreover, the thickness of the fiber capsule of the PVC was 253 ± 23 μm (Supplementary Fig. 18d). In contrast, the thickness of the pCBM/pSB capsule was much lower (61 ± 14 μm). Macrophage activation is considered an important factor in the inflammatory response, which is reflected by the distribution of CD68-positive cells. The inflammatory response was further evaluated by quantifying the number of macrophages accumulated around the sample. The number of macrophages in the tissues surrounding pCBM/pSB was 45.7%, which was less than that of PVC (68.8%) (Supplementary Fig. 18c, e). This was probably ascribed to the strong hydration ability of the pCBM/pSB hydrogel coating to resist nonspecific protein adsorption and avoid recognition by macrophages, further mitigating the tissue response in vivo.

### Antithrombogenicity of pCBM/pSB hydrogel coatings in vitro and ex vivo

**Fig. 6:**
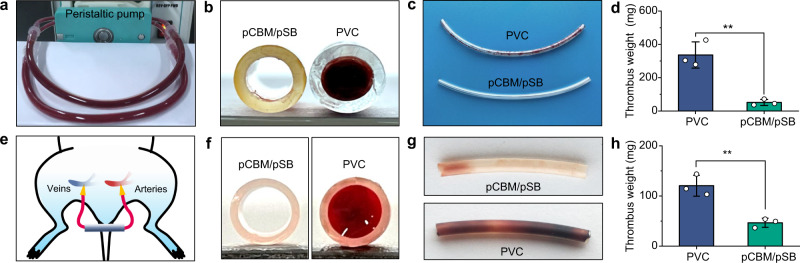
Antithrombogenicity of the pCBM/pSB hydrogel coatings in vitro and ex vivo blood circulation in rat model. **a** Scheme of circulation calcified whole blood in vitro. **b** Cross-section, (**c**) photographs, and (**d**) thrombus weight of circuits of PVC tubing and pCBM/pSB hydrogel coating modified PVC tubing after circulation for 1 h (*n* = 3). **e** Scheme of an ex vivo perfusion experiment in SD rats. **f** Cross-section, (**g**) photographs, and (**h**) thrombus weight of circuits of PVC tubing and pCBM/pSB hydrogel coating modified PVC tubing after circulation for 1 h without anticoagulants (*n* = 3). Data presented as mean ± SD and analyzed using a one-way ANOVA with Tukey’s post hoc test in (**d**, **h**), **P < 0.01. **d** *P* = 0.0036 (PVC vs pCBM/pSB, in vitro blood circulation), **h** *P* = 0.0049 (PVC vs pCBM/pSB, ex vivo blood circulation). Source data are provided as a Source Data file.

To evaluate the anticoagulant properties of the pCBM/pSB hydrogel coating, we used a peristaltic pump to build a dynamic circulation model of calcified whole blood (Fig. 6a). Pristine PVC tubing was severely occluded after 1 h of circulation, and many adherent clots formed on the inner wall (Fig. 6b, c). In contrast, the PVC tubing with the pCBM/pSB hydrogel coating remained unobstructed, and no thrombus could be observed. The quantitative results further showed that the thrombus weight on the PVC tubing was 336 ± 78 mg, while it was significantly reduced to 51 ± 18 mg after coating with the pCBM/pSB hydrogel (Fig. 6d). These results indicated that the pCBM/pSB hydrogel coating could prevent the formation of blood clots. The anticoagulant properties of the pCBM/pSB hydrogel coating were investigated by an ex vivo perfusion experiment in a rat (Fig. 6e). After 1 h of isolated circulation, the pristine PVC tubing was severely occluded, while the PVC tubing with the pCBM/pSB hydrogel coating remained completely unblocked (Fig. 6f, g). In addition, large amounts of adherent fibrin, activated platelets, and red blood cells were observed on pristine PVC tubing. In contrast, only a small number of platelets were found on the pCBM/pSB hydrogel coating modified PVC tubing (Supplementary Fig. 19). The thrombus weight was correspondingly reduced from 120 ± 20 mg of PVC to 46 ± 9 mg (Fig. 6h).

**Fig. 7:**
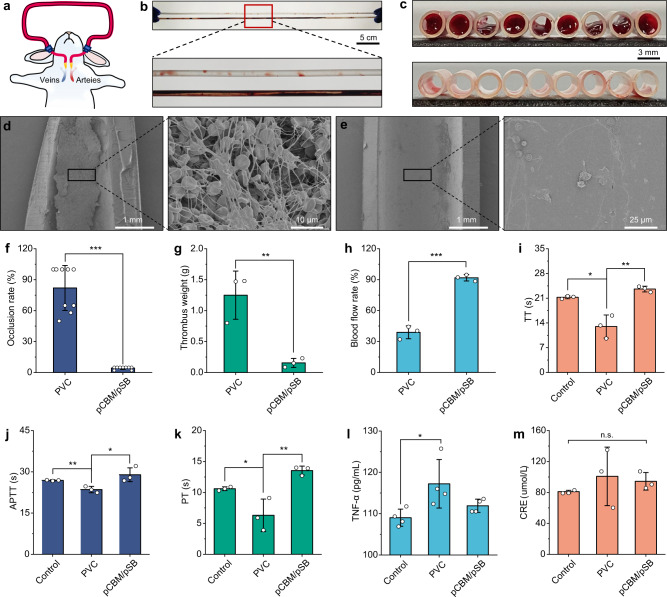
Antithrombogenicity and biochemical analysis of the pCBM/pSB hydrogel coatings in the rabbit model. **a** Scheme of the New Zealand white rabbits veno-venous extracorporeal circuit. **b** Photographs, (**c**) cross-section, SEM images of (**d**) PVC tubing, and (**e**) pCBM/pSB hydrogel coating modified PVC tubing after 2 h of circulation. Measurements in (**d**, **e**) were repeated three times independently with similar results. The quantitative results of **f** occlusion rate (*n* = 9), (**g**) thrombus weight (*n* = 3), (**h**) blood flow rate (*n* = 3), coagulation test of (**i**) TT (thrombin time) (*n* = 3), (**j**) APTT (activated partial thromboplastin time) (*n* = 3), and (**k**) PT (prothrombin time) (*n* = 3). The inflammatory cytokines level of (**l**) TNF-α (tumor necrosis factor-α) (*n* = 4) and (**m**) creatinine (CRE) (*n* = 3) in PVC tubing with/without pCBM/pSB hydrogel coating at the end of the circulation experiments. Data presented as mean ± SD and analyzed using a one-way ANOVA with Tukey’s post hoc test in (**f**–**m**), *P< 0.05, **P < 0.01, ***P < 0.001, n.s.: no significant difference at *P* > 0.05. **f** *P* < 0.001 (PVC vs pCBM/pSB, occlusion rate). **g** *P* = 0.0087 (PVC vs pCBM/pSB, thrombus weight). **h** *P* =  0.0121 (PVC vs pCBM/pSB, blood flow rate). **i** *P* = 0.0055 (control vs PVC, APTT), *P* = 0.0002 (PVC vs *p*CBM/pSB, APTT). **k** *P* = 0.0076 (control vs PVC, PT), *P* = 0.0365 (PVC vs pCBM*/*pSB, PT). **l** *P* = 0.0478 (control vs PVC, TNF-α), *P* = 0.0099 (PVC vs *p*CBM/pSB, TNF-α). **m** *P* = 0.4031 (control vs PVC, CRE), *P* = 0.7922 (PVC vs pCBM*/*pSB, CRE), *P* = 0.1038 (control vs pCBM/pSB, CRE). Source data are *p*rovided as a Source Data file.

To further evaluate the antithrombotic properties of the pCBM/pSB hydrogel coating in a setting closer to clinical applications. Commercial PVC tubing with/without pCBM/pSB hydrogel coatings (length of 50 cm and diameter of 3 mm) was assembled into the New Zealand white rabbits arteriovenous shunt model (Fig. 7a). After 2 h of isolated blood circulation, all samples were collected and the blockage rate, thrombus weight, and blood flow in the circuit were evaluated. A large number of thrombi formed in the pristine PVC tubing (Fig. 7b, c), which induced significant occlusive thrombosis (82.0 ± 21.7%). The pCBM/pSB hydrogel coating modified PVC tubing had almost no detectable occlusion (4.3 ± 1.3%) (Fig. 7f). A very serious clot could be clearly observed, which contains a cross-linked dense fiber network of fibrin, activated platelets, and red blood cells (Fig. 7d). However, only a few platelets and red blood cells were observed on the pCBM/pSB hydrogel coating surface. Moreover, these adhered platelets remained contracted or even round, suggesting that the adherent platelets were in an inactive state. (Fig. 7e). The thrombus weight on the pCBM/pSB hydrogel coating was reduced 8 times compared to that on PVC tubing (Fig. 7g). In addition, the blood flow rate in pristine PVC tubing was significantly reduced to 38.9 ± 6.2% compared with that at the initial rate, while the blood flow rate in pCBM/pSB hydrogel coating modified PVC tubing could still reach 91.8 ± 3.1% (Fig. 7h). This might be attributed to the strong hydration layer on the surface of the pCBM/pSB hydrogel coating, which reduced the adhesion of protein and cells. In addition, a lower surface friction coefficient was beneficial to improve patency.

The physiological and biochemical evaluations, including routine blood tests, coagulation, inflammation, and organ function were investigated after the end of the blood circulation experiment. The routine blood results showed that the red blood cell, hemoglobin, and platelet counts were decreased in the pSB and pCBM/pSB hydrogel groups compared to the control groups (Supplementary Fig. 20a–c). This was attributed to bleeding and platelet adhesion to the surface where the tubing interfaces. Furthermore, the coagulation tendency occurred when the blood was in contact with the pristine PVC tubing for a long time, which manifested as a significant shortening of TT, APTT, and PT (average 13.0, 23.5, and 6.3 s) (Fig. 7i–k). In contrast, the TT, APTT, and PT values of the pCBM/pSB hydrogel coating were extended to 23.6, 13.5, and 28.9 s, respectively (*P* < 0.05 or *P* < 0.01). In addition, the pCBM/pSB hydrogel coating can reduce the inflammatory response. The expression level of TNF-α in the PVC tubing increased significantly, indicating that the PVC tubing tended to induce inflammation. While the expression level of TNF-α in the pCBM/pSB hydrogel coating group was decreased (Fig. 7l). Finally, the values of liver enzyme alanine aminotransferase (ALT) and renal parameter creatinine (CRE) in all groups were detected within the normal range, suggesting that the pCBM/pSB hydrogel coating modified PVC tubing showed low organ toxicity within the 2 h blood circulation (Fig. 7m and Supplementary Fig. 20d).

## Discussion

In summary, we developed a poly(carboxybetaine) microgel-reinforced poly (sulfobetaine) (pCBM/pSB) hydrogel as a coating material for blood-contacting biomedical devices. The prepared pCBM/pSB hydrogel coatings were firmly bonded to the PVC substrate by network entanglement interactions between PVC and pSB. Moreover, the pSB as a continuous phase was integrated through the dispersed phase of pCBM to reinforce the mechanical strength of the pCBM/pSB hydrogel coatings. These coating could be easily formed on the sheet substrate surface and inner wall of long tubing. The obtained pCBM/pSB hydrogel coatings showed significant inhibition of fibrinogen, L929 cells, platelets, and bacterial adhesion. In addition, the pCBM/pSB hydrogel coating displayed controllable thickness, excellent stability and long-term resistance to biofilm formation properties. Importantly, the pCBM/pSB hydrogel coatings could not only mitigate the foreign body response but also prevent thrombus formation ex vivo in SD rats and rabbits blood circulation. Therefore, we envision that the microgel-reinforced zwitterionic hydrogel coating is a promising candidate to decrease the thrombosis of blood-contacting biomedical devices.

## Methods

### Materials

Commercial polyvinyl chloride (PVC) tubing (inner diameter, 5 mm or 3 mm), sheets (0.2  × 10  × 10 mm), and plates (2  × 30  × 30 mm) were purchased from Tianjin Huiman Technology Co., Ltd. (Tianjin, China). [2-(Methacryloyloxy) ethyl] dimethyl-(3-sulfopropyl) ammonium hydroxide (SBMA, 95%), *N, N*’-methylenebisacrylamide (MBA, 99%), Span 80, Tween 80, n-hexane, tetrahydrofuran, 2-hydroxy-2-methylpropiophenone (photoinitiator 1173, 97%), and benzophenone (BP, 99%) were received from Aladdin (Shanghai, China). Fibrinogen from human plasma (≥80% of protein is clottable) and lysozyme (protein ≥90%) were supplied by Sigma-Aldrich (USA). Other chemicals were analytical grade without purification unless otherwise indicated.

### Preparation of pCBM and pCBM/pSB hydrogel

2-Carboxy-*N, N*-dimethyl-*N*-(3′-acrylamidopropyl) ethanaminium inner salt (CBAA) was synthesized according to previous reports^36,37^. pCB microgel (pCBM) was prepared by inverse miniemulsion polymerization. First, 2.28 g of CBAA, MBA (1 mol%, 4 mol%, and 7 mol% relatives to CBAA), and APS (0.1 mol% relative to CBAA) were dissolved in 5 mL of H_2_O. Then, 0.56 g of Tween 80 (0.44 mmol) and 1.72 g of Span 80 (4 mmol) were dissolved in 100 mL of n-hexane as the oil phase. The CBAA aqueous solution was added dropwise to the oil phase and treated by ultrasound in an ice bath for 10 min. A stable emulsion was obtained. The emulsion was heated to 70 °C and polymerization of CBAA was carried out in a nitrogen atmosphere. After 4 h, the reaction solution was cooled to room temperature and the microemulsion was thoroughly broken by adding 80 mL of tetrahydrofuran. The obtained precipitate was washed three times with 50 mL of tetrahydrofuran to remove the unreacted monomers. Finally, pCBM was obtained via dialysis and freeze-drying.

To prepare the pCBM/pSB hydrogel, a proper amount of dry pCBM was fully swelled in SBMA aqueous solution (4 M) containing 0.1 mol% MBA and 0.01 mol% photoinitiator 1173. The obtained paste-like pCBM/pSB was deoxidized with argon for 1 h and then transferred to a tubular mold. After deoxygenation with argon for 1 h, the pre-gel solution was then transferred to a 2.5 mL cylindrical mold (8.5 mm in diameter) or a rectangular mold (10 cm × 10 cm × 1 mm). The pCBM/pSB hydrogels were prepared by initiating radical polymerization under UV irradiation (302 nm, 6 W) for 6 h. The resulting pCBM/pSB hydrogels were soaked in water for 48 h to remove unreacted reagents. The zwitterionic pSB hydrogels without pCBM were prepared using the same method as the control. The pCBM/pSB hydrogels were denominated as pCBMx/pSB, where x (10, 20, and 30 mg/mL) was the concentration of pCBM.

### Mechanical performance tests

The mechanical properties of the pCBM/pSB hydrogel were measured by a universal electromechanical tester (WDW-05, Si Pai Inc, China) at room temperature. For compression tests, cylindrical pCBM/pSB hydrogels with 8.5 mm in diameter and 8.5 mm in height were investigated at a fixed rate of 5 mm/min. The compressive strain limit was set to 95%. For the tensile test, a pCBM/pSB hydrogel sheet with a thickness of 1 mm was cut into strips with a length of 30 mm, and a width of 10 mm, and the tensile rate was fixed at 50 mm/min. The shape recovery characteristics were evaluated by 100 consecutive loading and unloading cycles in the strain range of 0–70%. In addition, to avoid the evaporation of water during the test, silicone oil was used to coat the surface of the pCBM/pSB hydrogel.

### Swelling behaviors tests

The swelling ratio (SR) of pCBM/pSB hydrogels was determined by the gravimetric method. First, the cylindrical hydrogel (diameter 8.5 mm, height 5 mm) was immersed in PBS at room temperature to achieve swelling equilibrium, and the wet weight (M_w_) of the pCBM/pSB hydrogel was recorded. The dry weight (M_d_) of the pCBM/pSB hydrogel was recorded after freeze-drying. The SR of pCBM/pSB hydrogel was calculated as follows

1 $${{{{{\rm{SR}}}}}}=\frac{{M}_{w}-{M}_{d}}{{M}_{d}}\times 100\%$$

### Preparation of pCBM/pSB hydrogel coatings

The pristine PVC sheet was first treated with an atmospheric plasma cleaner for 3 min and then immersed in acetone containing 5 wt% benzophenone for 5 min. The PVC sheet was washed alternately with ethanol and water and dried under vacuum, denominated PVC-BP. A pre-gel solution containing 4 M SBMA, MBA (0.1 mol%, relative to SBMA), photoinitiator 1173 (0.01 mol%, relative to SBMA), and 20 mg/mL pCBM was poured onto the surface of the PVC-BP substrate. The pre-gel solution was injected via a 10 mL syringe onto a PVC-BP substrate containing a silicone spacer (3 cm × 3 cm or 5 cm × 5 cm). Subsequently, the acrylic sheet was covered and clamped with tweezers. The pCBM/pSB hydrogel coating was formed on the PVC-BP substrate under argon protection after UV lamp (302 nm, 6 W) irradiation for 6 h. The prepared pCBM/pSB hydrogel coating was soaked in deionized water for 48 h to remove unreacted reagents. For the flat PVC surface, the height of the pre-gel solution was adjusted using silicone spacers of different thicknesses (0.1 mm and 1 mm) to achieve the regulation of the thickness of the pCBM/pSB hydrogel coating. For PVC tubing, the pCBM/pSB pre-gel solution should completely fill the lumen, and the pre-solution formulation without hydrophilic photoinitiator 1173. Therefore, during the UV polymerization process, the swollen initiator on the surface of the PVC tubing polymerizes a second polymer network. This network penetrated the first network of pCBM and resulted in the uniform coating of an ultrathin and uniform hydrogel layer on the device. In addition, the pSB hydrogel coating was also prepared on a PVC surface using a similar method without pCBM. All obtained hydrogel coatings were rinsed with deionized water for 30 min to remove unreacted reagents.

### Characterization of pCBM/pSB hydrogel coating

The surface chemical composition of the pristine PVC substrate, pSB hydrogel coating, and pCBM/pSB hydrogel coating was analyzed by attenuated total reflectance Fourier transform infrared spectroscopy (ATR-FTIR, Nicolet 6700) and X-ray photoelectron spectroscopy (XPS, Thermo Fisher Scientific). Scanning electron microscopy (SEM, Regulus 8100 and S-4800, Hitachi) was used to observe the surface morphology. The thickness of the pCBM/pSB hydrogel coating was measured using a fluorescence microscope (ECLIPSE Ts2 Nikon). The hydrophilicity of the pCBM/pSB hydrogel coating was characterized by contact angle measurement (JC2000FM, Shanghai Zhongchen Digital Technology Instrument).

### Antifouling property evaluation

The adhesion behaviors of proteins, cells, platelets, and bacteria on the surface of pristine PVC sheets, pSB hydrogel coatings, and pCBM/pSB hydrogel coatings were investigated to evaluate the antifouling properties. For protein adsorption, the samples were incubated in a protein solution (5 mg/mL of Fibrinogen and 10 mg/mL of lysozyme) at 37 °C for 2 h. Then, the sample was washed three times with PBS and soaked in SDS solution (2 wt%). After being treated by ultrasound for 30 min, protein adsorption was measured with a microplate reader at 562 nm according to the instructions of the BCA micro-detection kit. For cell adhesion behaviors, mouse ftibroblasts (L929) cells (Cat. No.: iCell-m026) lines were purchased from iCell Bioscience (Shanghai, China). L929 cells (1 × 10^5^) with green fluorescent markers were seeded on the sample surface. After being cultured for 24 h, all the samples were washed with PBS to remove unattached cells. The morphology of the adherent cells on the sample surface was observed with a fluorescence microscope. In addition, the cell viability of adherent cells was tested using the MTT cell proliferation and cytotoxicity assay kit (Cat. No.: ST1537, Biyuntian). For platelet adhesion behaviors, the samples were incubated in 200 μL of platelet-rich plasma (PRP) for 2 h at 37 °C. The activity was measured via lactic dehydrogenase (LDH) assay at 450 nm and the morphology of the adhered platelets was investigated by SEM. For bacteria adhesion behaviors, the Gram-negative *Escherichia coli* (*E. coli*, ATCC25922) and Gram-positive *Staphylococcus aureus* (*S. aureus*, ATCC6538) were obtained from Shanghai Luwei Technology Co., Ltd. (Shanghai, China). In short, 1 mL of the microbial suspension was dropped on the sample surface in a 24-well plate and cultured at 37 °C for 4 h. After washing with PBS, the samples were immersed in 2 mL of PBS solution and treated by ultrasonic treatment. 100 μL of the resulting solution was spread on Luria-Bertani agar plates, and the number of colonies in each sample was calculated after 12 h of incubation. In addition, the bacteria morphology was observed by SEM.

### Stability test of pCBM/pSB hydrogel coatings

The adhesion strength between the pCBM/pSB hydrogel coating and PVC sheet was evaluated by a peel test. The prepared pCBM/pSB hydrogel coating on PVC were glued to a flexible and non-extensible film through commercial cyanoacrylate glue. The 90°-peeling experiment was performed at a 10 mm/min peeling rate with a 50 N load cell. To investigate the stability of the pCBM/pSB hydrogel coating on PVC in a dynamic microenvironment, first, the pCBM/pSB hydrogel coating and pSB hydrogel coating on PVC were fixed on the inner wall of the beaker (*d* = 7 cm). PBS solution (100 mL) was added and a continuous shearing (35.2 g) was applied. The contact angle of the samples was investigated after 7 d, 14 d, and 21 d. The antibacterial adhesion properties were investigated after 7 d. Second, the stability of the pCBM/pSB hydrogel coating was evaluated by impacting with water flow for 0.5 h. The velocity of the water flow was 45.6 mL/s, and the distance of the sample (3  × 3  × 3 cm) to the water source was 0.5 m. The adhesion strength of the pCBM/pSB hydrogel coating on PVC before and after flushing was investigated. Third, for the sandpaper abrasion test, all samples were placed on 400 mesh SiC sandpaper and rubbed 5 cm in the horizontal direction under a weight of 500 g. This process was repeated 100 times. The friction coefficient of the samples was measured via the coefficient of friction tester. Fourth, the mechanical stability of the pCBM/pSB hydrogel coating was tested through a bending experiment in water. After 1000 cycles, the integrity of the pCBM/pSB hydrogel coating was investigated by SEM.

The long-term antifouling ability of the pCBM/pSB hydrogel coating was determined by observing the formation of biofilms. After UV sterilization, the pristine PVC and pCBM/pSB hydrogel coated PVC sheets were placed in culture medium with a high concentration of bacteria (1 × 10^7^ CFU/mL of *E. coli* and *S. aureus*). The 50% solution was replaced with fresh sterile Luria-Bertani medium every 2 d. After 14 d and 30 d of long-term incubation, all samples were rinsed with PBS, immersed in 2.5 wt% glutaraldehyde solution for 4 h, dehydrated in a series of gradient ethanol solutions, and dried in a vacuum. Finally, the formation of biofilms on the sample surface was observed using SEM.

### Cytotoxicity and hemolysis assay

The pristine PVC, pSB, and pCBM/pSB hydrogel coated PVC were immersed in an RPMI-1640 medium for 1 d and 60 d to obtain extraction. In total, 1 × 10^4^ of L929 cells were seeded in a 96-well plate and incubated overnight. Subsequently, the culture medium was replaced by the extractions. After incubation for 24 h, cell viability was evaluated by MTT cell proliferation and cytotoxicity assays kit. L929 cells cultured with RPMI-1640 medium served as controls. The cell viability was calculated as follows

2 $${{{{{\rm{Cell \, viability}}}}}}\;(\%)=\frac{{A}_{{{{{{\rm{sample}}}}}}}-{{{A}}}_{{{{{{\rm{blank}}}}}}}}{{A}_{{{{{{\rm{control}}}}}}}-{{{A}}}_{{{{{{\rm{blank}}}}}}}}\times 100$$

For the hemolysis test, whole blood was diluted with PBS at a ratio of 1:4 (v/v). Each sample was incubated in 2 mL of blood solution for 4 h at 37 °C. The solution was centrifuged at 120 × *g* for 15 min. The absorbance of the supernatant was measured at 540 nm by a microplate reader. Whole blood diluted in PBS and deionized water was used as a negative control and positive control, respectively. The percentage of hemolysis was calculated as follows

3 $${{{{{\rm{Hemolysis}}}}}}\,(\%)=\frac{{A}_{{{{{{\rm{sample}}}}}}}-{A}_{{{{{{\rm{negative}}}}}}}}{{A}_{{{{{{\rm{positive}}}}}}}-{A}_{{{{{{\rm{negative}}}}}}}}\times 100$$

### Subcutaneous implantation test

The inflammatory response of pCBM/pSB hydrogel coating was performed by implanting the samples into the back subcutaneous tissue of male Sprague Dawley (SD) rats. After 30 d, the implanted PVC sheet and pCBM/pSB hydrogel coated PVC sheet were collected and fixed with 4% paraformaldehyde for 24 h, followed by paraffin embedding and sectioning. To assess fibrosis formation upon hydrogel treatment, the samples were stained with Masson’s trichrome via standard protocols. The thickness of the fibrous capsule was measured using Image J software. Meanwhile, immunohistochemical staining was performed to evaluate the inflammatory reaction. The samples were then incubated with primary antibodies (1:1000 dilution) for a rabbit anti-rat CD68 (Abcam; Cat. No.: ab125212) to label macrophage cells overnight at 4 °C, followed by incubation with Cy3–conjugated Affinipure Goat Anti-Rabbit IgG (H + L) secondary antibodies (1:500 dilution) (Proteintech, Cat. No.: SA00009-2). The samples were counterstained with DAPI (Abcam, Cat. No.: ab104139) and imaged by a LSM 780 confocal microscope (Zeiss, Oberkochen, Germany).

### Antithrombogenicity test in vitro

A blood circulation device was prepared according to our previous report^33^. First, PVC tubing with/without pCBM/pSB hydrogel coatings was rinsed three times with 75% alcohol, sterile saline, sodium citrate, and sterile saline, respectively. In all, 10 mL of fresh rabbit blood containing anticoagulant was injected into the interior of the tubing, and then connected to the end to close into a ring. To eliminate the interference of anticoagulants, 0.2 M CaCl_2_ solution in normal saline was injected into the anticoagulant rabbit blood to accelerate thrombus formation. Blood flow was set to 0.2 L/min by the peristaltic pump. The circuit was stopped until the pristine PVC tubing was clotted. All the samples were washed with PBS, photographed, and weighed.

### Antithrombogenicity test ex vivo

The ex vivo antithrombogenicity behaviors of the pCBM/pSB hydrogel coating were investigated using an extracorporeal circuit (ECC) model of SD rats and New Zealand white rabbits. For the SD rats ECC model, an artery-vein extracorporeal circuit was established by cannulating the left artery and the right femoral vein. SD rats were anesthetized by intraperitoneal injection of pentobarbital (15–20 mg/kg), and the abdominal aorta and celiac vein were separated and fixed using medical sutures. Indwelling needles were attached to both sides of the PVC tubing with/without pCBM/pSB hydrogel coatings and inserted into the abdominal aorta at an oblique 30° angle. The abdominal aorta was clamped with a hemostat after blood had flowed from one end of an indwelling needle. Another indwelling needle was inserted into the abdominal vena cava to form the blood circulation circuit of the abdominal aortic vein in SD rats. After 1 h of blood circulation, the circuit was stopped and removed from the animal, and rinsed with PBS. The residual thrombus in the tubing was collected, photographed, and weighed. In addition, all samples were fixed with glutaraldehyde solution and observed by SEM.

For the New Zealand white rabbits ECC model experiment, an artery-vein extracorporeal circuit was established by cannulating the left carotid artery and the right jugular vein. New Zealand white rabbits (2.5–3.5 kg) were anesthetized by injection of pentobarbital (15–20 mg/kg). PVC tubing with/without pCBM/pSB hydrogel coating (length: 50 cm, diameter: 3 mm) were connected to the indwelling needle at both ends. After separating the carotid artery and venous blood vessel, the indwelling needle was inserted into the arterial vessel of the rabbit neck at an oblique 30° angle, and the carotid artery was clamped with hemostatic forceps. The indwelling needle at the other end was inserted into the venous blood vessel of the rabbit neck in the same way. Then the hemostatic forceps were released to form the blood circulation circuit of the artery and vein in the neck of the New Zealand white rabbit. After the blood circulation started, the inside of the tubing was observed for blockage every 15 min. After 2 h, the weight of residual thrombus on the samples was counted. Afterward, all the samples were fixed and dehydrated for SEM observation. Blood samples were analyzed using a Mindray BC-5100 automatic hematology analyzer (Mindray Corp., Shenzhen, China) to obtain red blood cell (RBC), hemoglobin, and platelet counts. The concentrations of TNF-α (tumor necrosis factor-α) in the animal plasma samples were measured at the beginning and end of the experiments by Rabbit TNF-α DuoSet ELISA Kit (R&D Systems, Cat. No.: DY5670). The activated partial thromboplastin time (APTT), Thrombin time (TT), and prothrombin time (PT) were measured by an automatic blood coagulation analyzer CA-50 (Sysmex Corporation, Kobe, Japan). Moreover, alanine aminotransferase (ALT), and creatinine (CRE) were determined by an automatic biochemical analyzer (7180, Hitachi, Japan).

### Statistical analysis

All data are presented as the mean ± SD. Statistical analysis was performed using Origin 2021 software and Microsoft Excel 2016. The value of *P* < 0.05 was considered significant. n.s.: no significant difference at *P* > 0.05.

### Ethical issues on animal experiments

The animal experiments were performed in accordance with the Guidelines for Care and Use of Laboratory Animals of the Tianjin Medical University General Hospital and approved by the Animal Ethics Committee of the Tianjin Medical University General Hospital (Tianjin, China).

### Reporting summary

Further information on research design is available in the Nature Research Reporting Summary linked to this article.

## Supplementary information

Supplementary Information

Reporting Summary

ChemDraw files

## Data Availability

All data generated for this study are included in the main and supplementary figures. Other data that support the findings of this study are available on request from the corresponding author.  Source data are provided with this paper.

## Source data

Source Data
