# Supplementary material for: Microgel reinforced zwitterionic hydrogel coating for blood-contacting biomedical devices
Source: Nat Commun. 2022 Sep 12;13:5339. doi: 10.1038/s41467-022-33081-7 (PMC9468150; doi:10.1038/s41467-022-33081-7)
Supplement: Supplementary file 1 — Supplementary Information [file 41467_2022_33081_MOESM1_ESM.pdf]

Supplementary Information for

Microgel reinforced zwitterionic hydrogel coating for blood-contacting biomedical devices

Mengmeng Yao[1,2#], Zhijian Wei[3,4,5#], Junjin Li[3,4,5], Zhicheng Guo[1], Zhuojun Yan[1], Xia Sun[1], Qingyu Yu[1], Xiaojun Wu[1], Chaojie Yu[1], Fanglian Yao[1,2,6], Shiqing Feng[3,4,5*], Hong Zhang[1,2*], Junjie Li[1,2*]

[1]School of Chemical Engineering and Technology, Tianjin University, Tianjin 300350, China.

[2]Frontiers Science Center for Synthetic Biology and Key Laboratory of Systems Bioengineering (Ministry of Education), Tianjin University, Tianjin 300350, China.

[3]International Science and Technology Cooperation Base of Spinal Cord Injury, Department of Orthopedic Surgery, Tianjin Medical University General Hospital, Tianjin, China.

[4]Department of Orthopaedics, Qilu Hospital, Cheeloo College of Medicine, Shandong University, Jinan, Shandong 250012, China.

[5]Shandong University Centre for Orthopaedics, Cheeloo College of Medicine, Shandong University, Jinan, Shandong, 250012, China.

[6]School of Materials Science and Engineering, East China Jiaotong University, Nanchang 330013, China.

# These authors contributed equally: Mengmeng Yao and Zhijian Wei.

*Corresponding author: Junjie Li, E-mail: li41308@tju.edu.cn, Hong Zhang, E-mail: zhanghong@tju.edu.cn, Shiqing Feng, E-mail: sqfeng@tmu.edu.cn

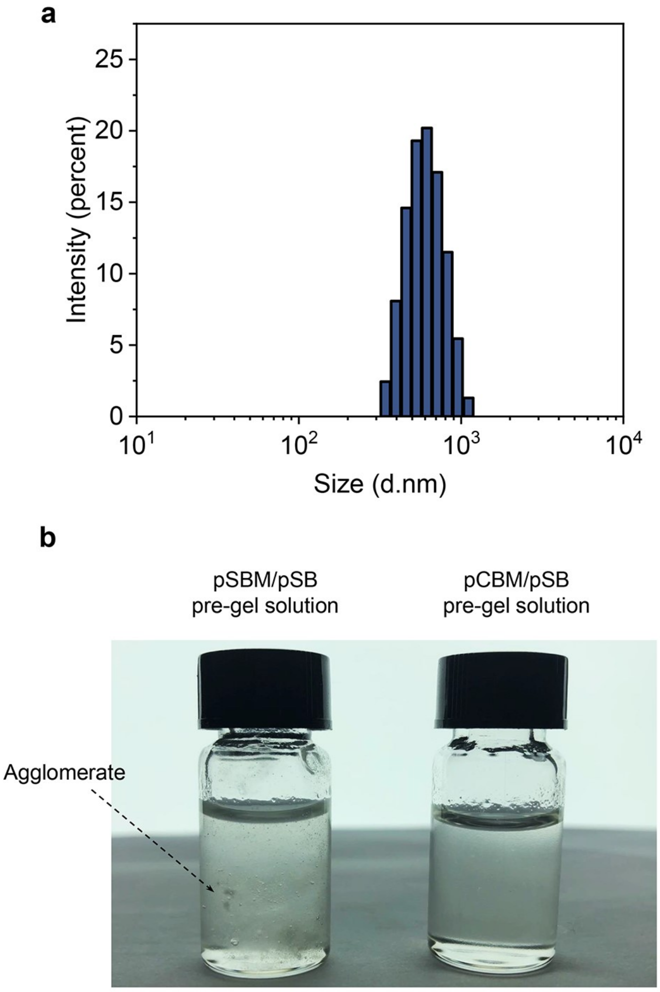

Supplementary Fig. 1. (**a**) Particle size distribution of pCBM. (**b**) Optical photo of pSBM/pSB and pCBM/pSB pre-gel solution.

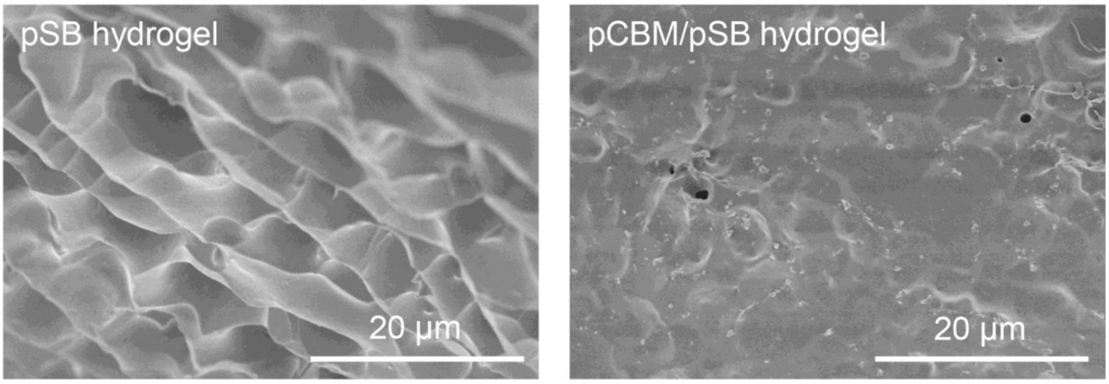

Supplementary Fig. 2. Cross-sections SEM images of pSB hydrogels and pCBM/pSB hydrogels. These measurements were repeated three times independently with similar results.

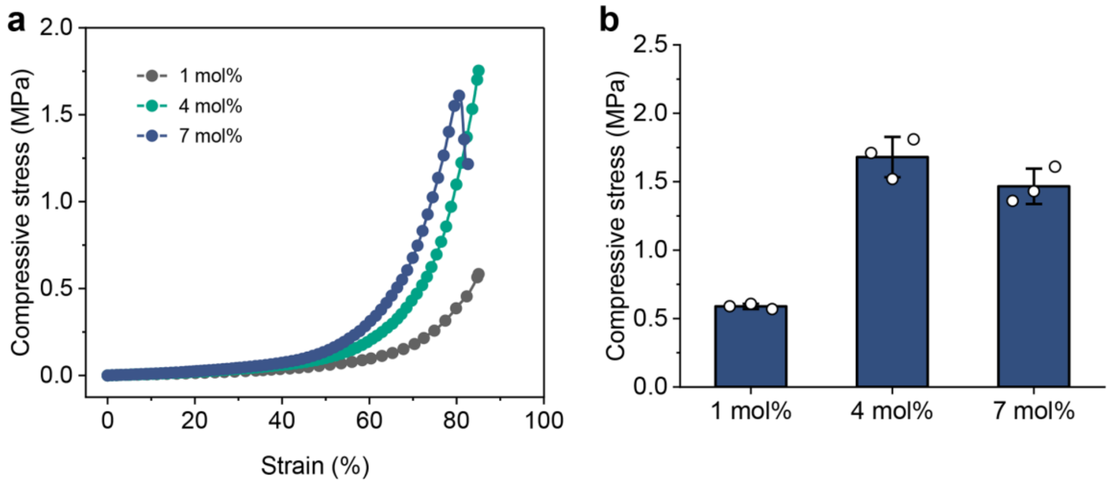

Supplementary Fig. 3. The effect of different MBA concentrations of pCBM on the (**a**) compressive stress-strain curves and (**b**) compressive stress of the pCBM/pSB hydrogel when the strain is 85% (n=3). Data presented as mean ± SD in **b**. Source data are provided as a Source Data file.

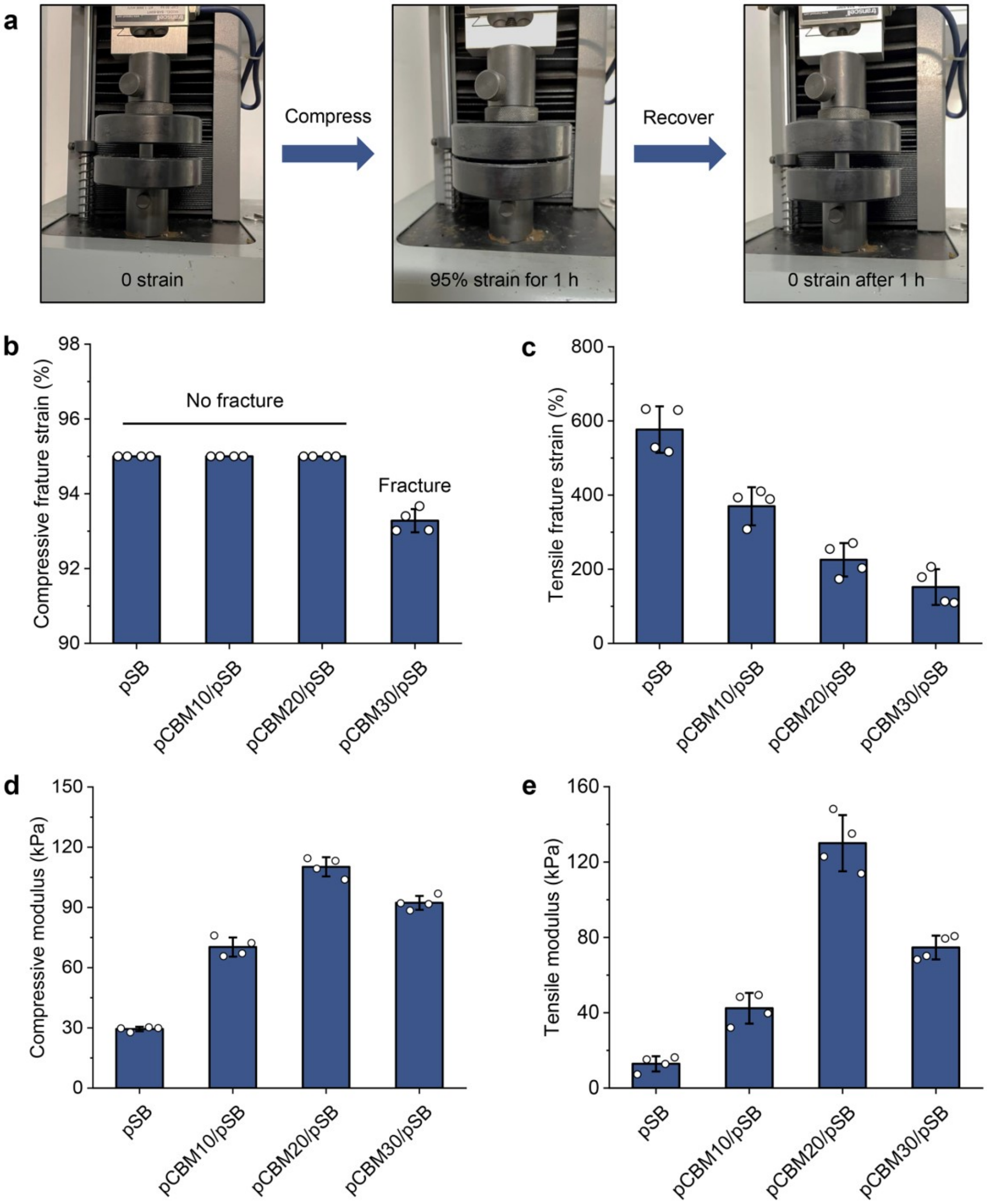

Supplementary Fig. 4. (**a**) The pCBM20/pSB hydrogel can sustain a high compression strain of 95% for 1 h and recover its original shape after relaxing. (**b**) Compressive (n=4) and (**c**) tensile fracture strain (n=4) of the pCBM/pSB hydrogel with varying pCBM concentrations. The effect of pCBM concentrations on the (**d**) compressive modulus (n=4) and (e) tensile modulus (n=4) of pCBM/pSB hydrogels. Data presented as mean ± SD in **b-e**. Source data are provided as a Source Data file.

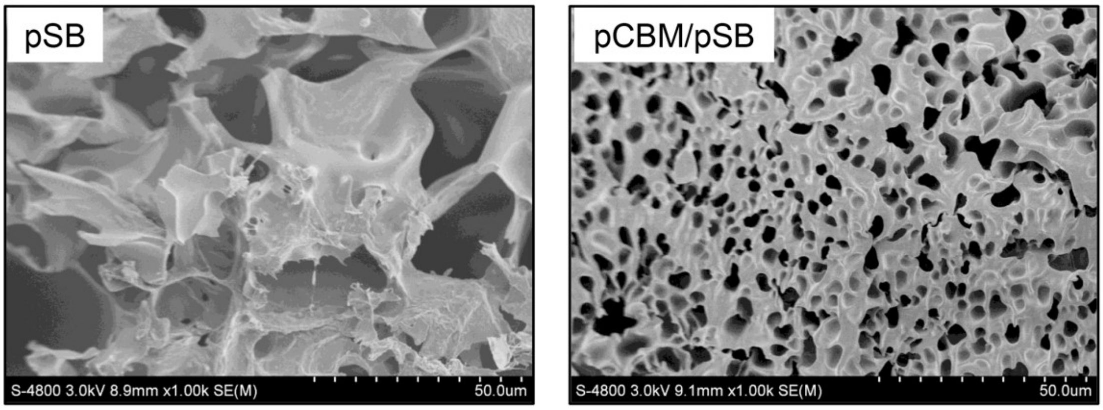

Supplementary Fig. 5. SEM image of the pSB and pCBM/pSB hydrogel coatings after swelling equilibrium in PBS. These measurements were repeated three times independently with similar results.

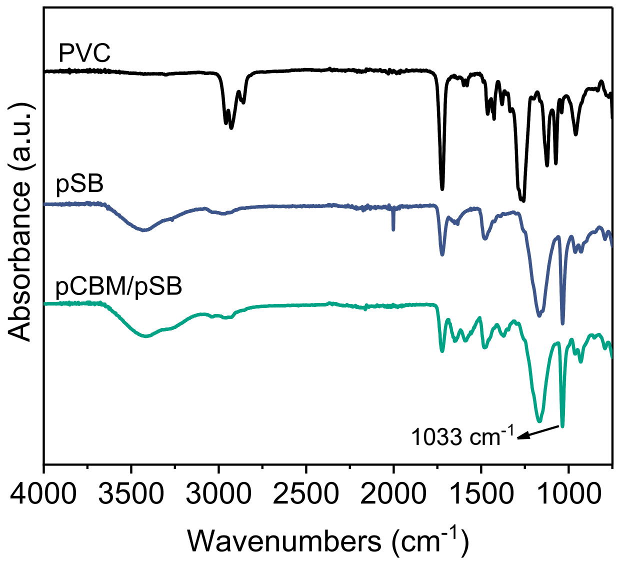

Supplementary Fig. 6. ATR-FTIR of PVC substrate, pSB hydrogel coating and pCBM/pSB hydrogel coating.

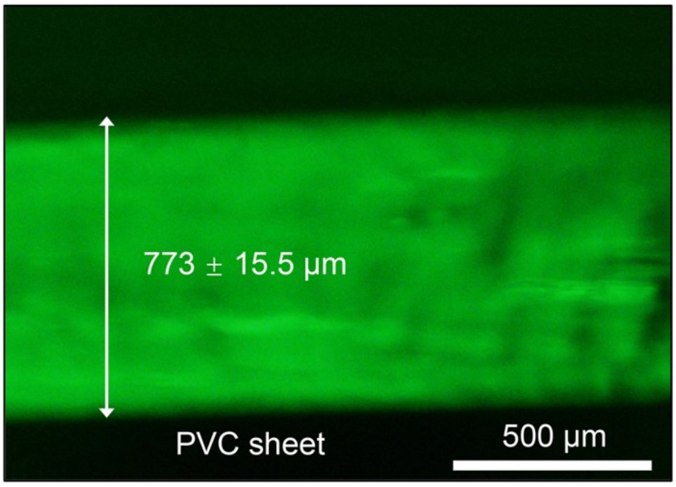

Supplementary Fig. 7. Fluorescence image of cross-section of the pCBM/pSB hydrogel coatings on PVC sheet by using 1 mm thick pre-gel solution. These measurements were repeated three times independently with similar results.

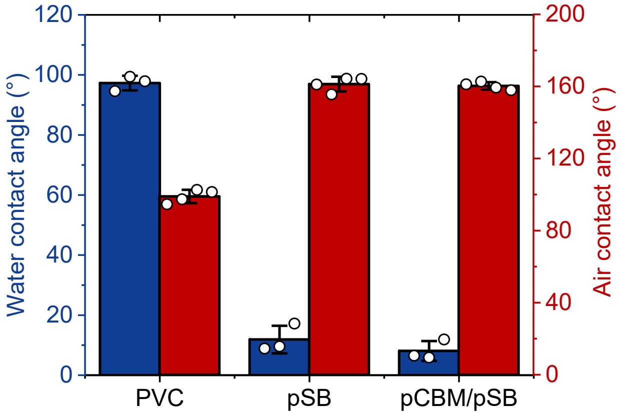

Supplementary Fig. 8. Water contact angle and air contact angle of PVC substrate, pSB hydrogel coating and pCBM/pSB hydrogel coating (n=4). Data presented as mean ± SD. Source data are provided as a Source Data file.

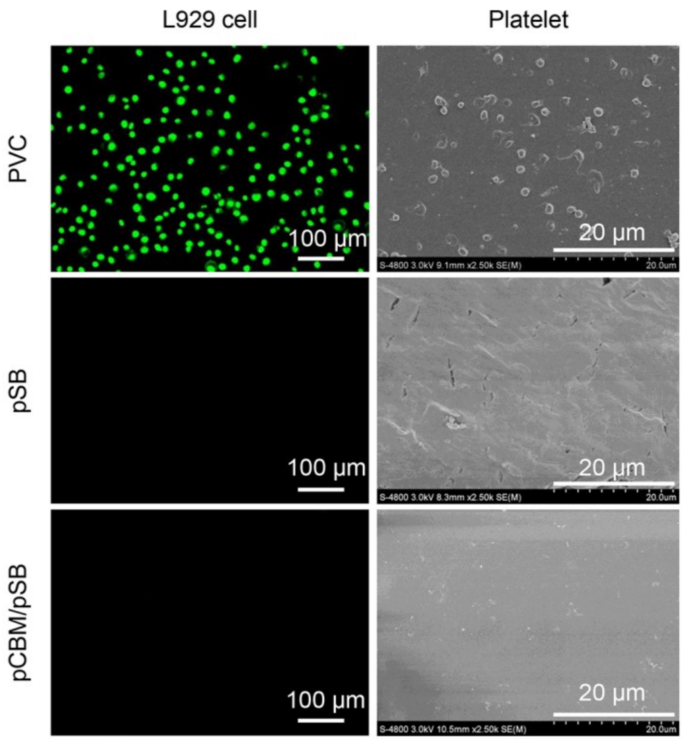

Supplementary Fig. 9. Fluorescence microscopy images of L929 cells and SEM images of platelets on PVC, pSB hydrogel coating, and pCBM/pSB hydrogel coating. These measurements were repeated three times independently with similar results.

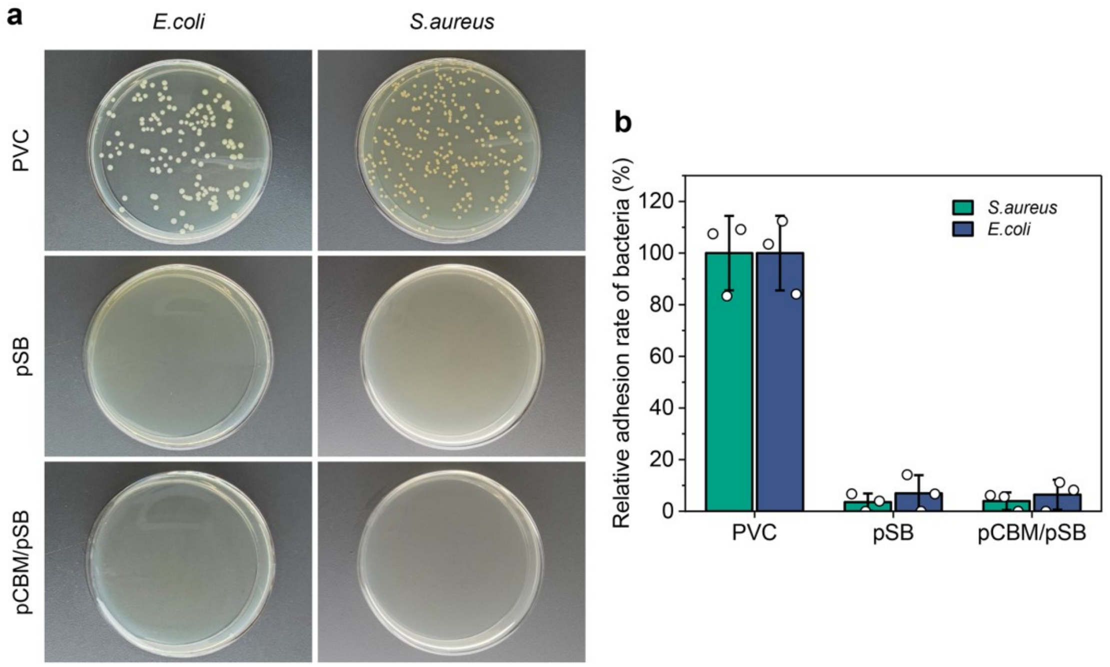

Supplementary Fig. 10. (**a**) Optical images of *E. coli* colonies and *S. aureus* colonies on PVC, pSB hydrogel coating, and pCBM/pSB hydrogel coating. (**b**) Relative adhesion rate of *E. coli* and *S. aureus* adhered on PVC, pSB hydrogel coating, and pCBM/pSB hydrogel coating (n=3). Data presented as mean ± SD in **b**. Source data are provided as a Source Data file.

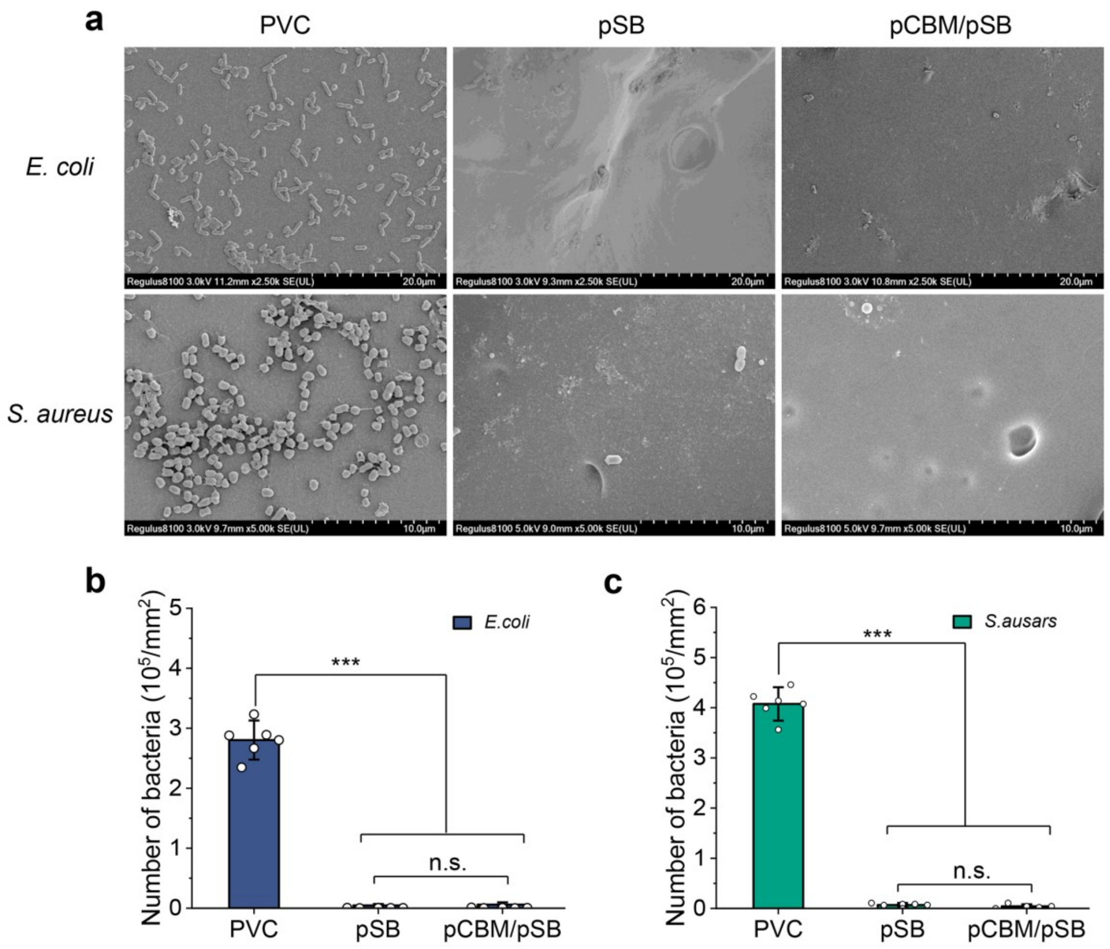

Supplementary Fig. 11. (**a**) SEM images of *Escherichia coli* (*E. coli*) and *Staphylococcus aureus* (*S. aureus*) on PVC, pSB hydrogel coating, and pCBM/pSB hydrogel coating. Measurements in **a** were repeated three times independently with similar results. Number of adherents of (**b**) *S. aureus* (n=6) and (**c**) *E. coli* (n=6) was calculated by Image J software. Data presented as mean ± SD and analyzed using a one-way ANOVA with Tukey's post hoc test in **b**-**c**, ***p < 0.001, n.s.: no significant difference at p > 0.05. **b** p < 0.001 (PVC vs pCBM/pSB, PVC vs pSB, *E. coli*), p = 0.2488 (pSB vs pCBM/pSB, *E. coli*). **c** p < 0.001 (PVC vs pCBM/pSB, PVC vs pSB, *S. aureus*), p = 0.4576 (pSB vs pCBM/pSB, *S. aureus*). Source data are provided as a Source Data file.

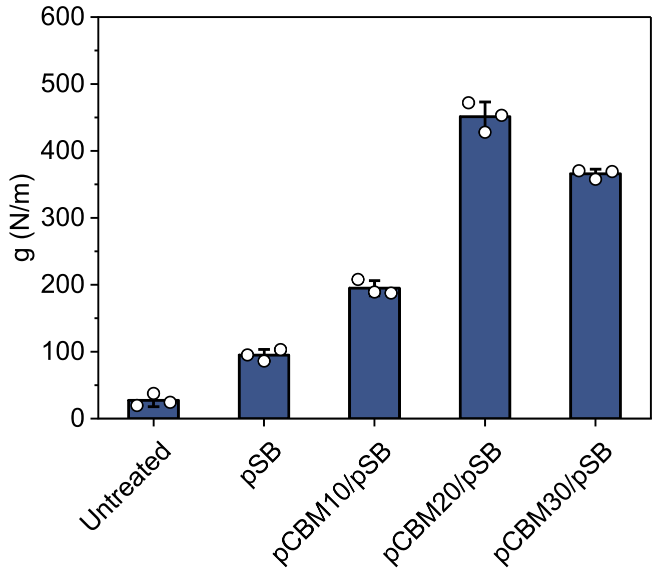

Supplementary Fig. 12. The peeling force per unit length of untreated, pSB, pCBM10/pSB, pCBM20/pSB, and pCBM30/pSB hydrogel coatings (n=3). Data presented as mean ± SD. Source data are provided as a Source Data file.

a i

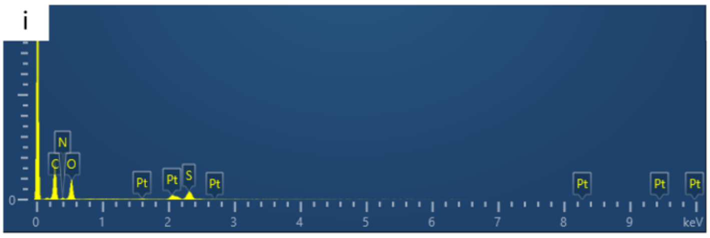

ii

Elements	Atom %
C	65.18
O	23.65
S	4.40
N	5.66
Pt	1.11

b i

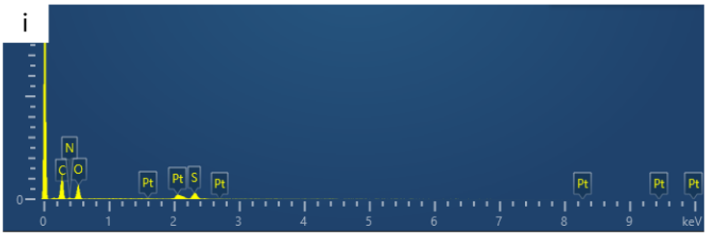

ii

Elements	Atom %
C	64.49
O	22.97
S	4.58
N	6.55
Pt	1.42

Supplementary Fig. 13. The EDS and atomic content of (**a**) the side of the PVC substrate and (**b**) the side of the peeling film after the peel test.

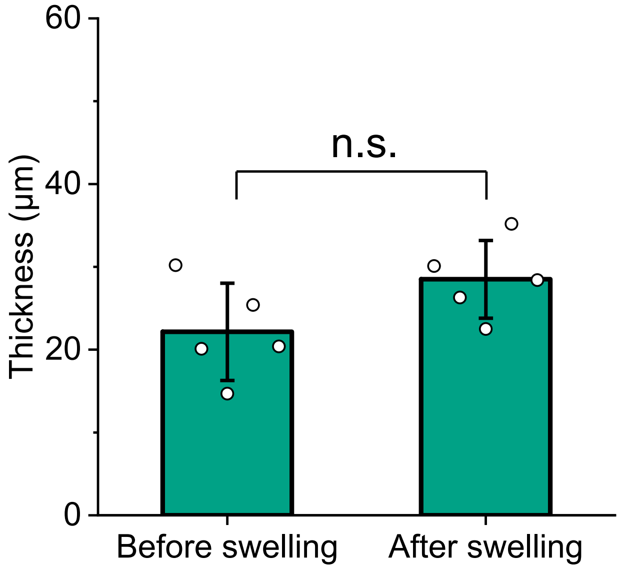

Supplementary Fig. 14. The thickness of pCBM20/pSB hydrogel coating before and after swelling (n=5). Data presented as mean ± SD and analyzed using a one-way ANOVA with Tukey's post hoc test, n.s.: no significant difference at $p > 0.05$. $p = 0.0963$ (Before swelling vs After swelling, Thickness). Source data are provided as a Source Data file.

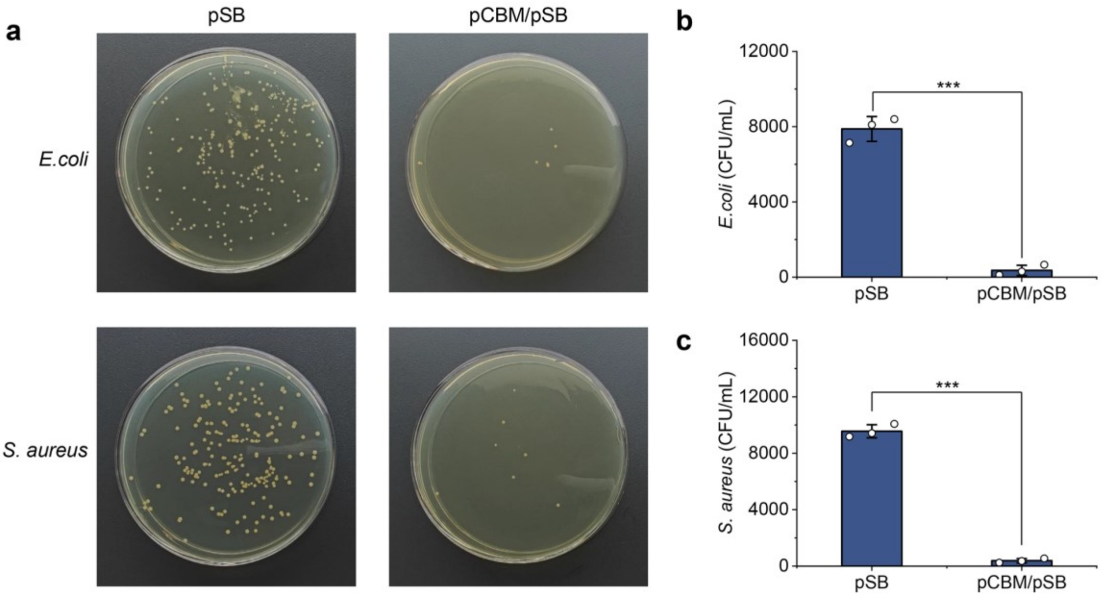

Supplementary Fig. 15. (**a**) Optical images of *E. coli* colonies and *S. aureus* colonies on the pSB and pCBM/pSB hydrogel coatings after shearing in PBS for 21 d. (**b**) Adhesion of *E. coli* colonies (n=3) and (**c**) *S. aureus* (n=3) colonies on the pSB and pCBM/pSB hydrogel coatings after shearing in PBS for 21 d. Data presented as mean ± SD and analyzed using a one-way ANOVA with Tukey's post hoc test in **b**-**c**, ***$p < 0.001$. **b** $p < 0.001$ (pSB vs pCBM/pSB, *E. coli*). **c** $p < 0.001$ (pSB vs pCBM/pSB, *S. aureus*). Source data are provided as a Source Data file.

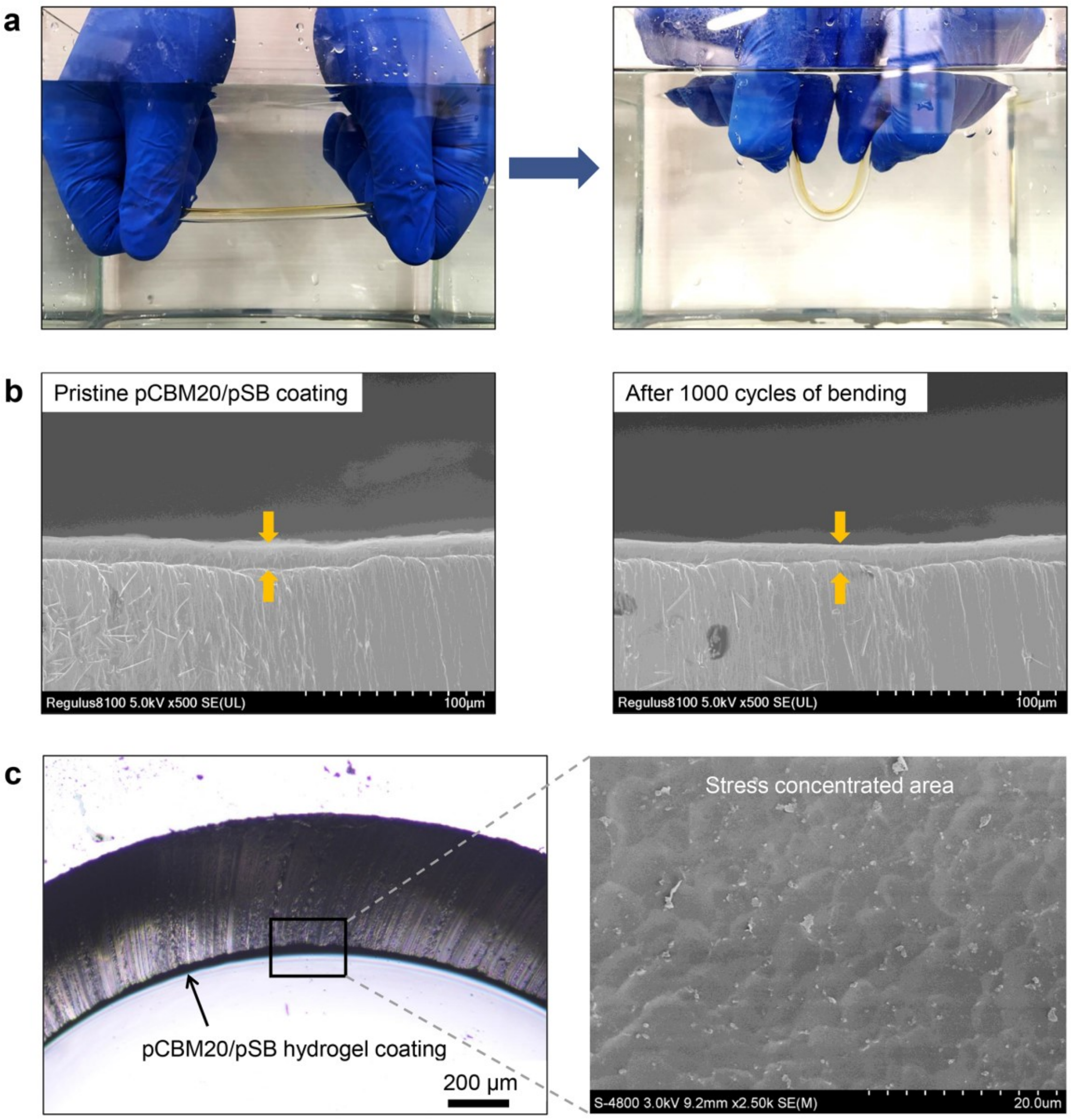

Supplementary Fig. 16. (**a**) Optical photographs and (**b**) SEM cross-sections of the pCBM20/pSB hydrogel coating before and after being bent 1000 times under water. Measurements in **b** were repeated three times independently with similar results. (**c**) Cross-section optical photo and SEM image (surface morphology) of the pCBM/pSB hydrogel coating after 1000 bending test. Measurements in **c** (right panel) were repeated three times independently with similar results.

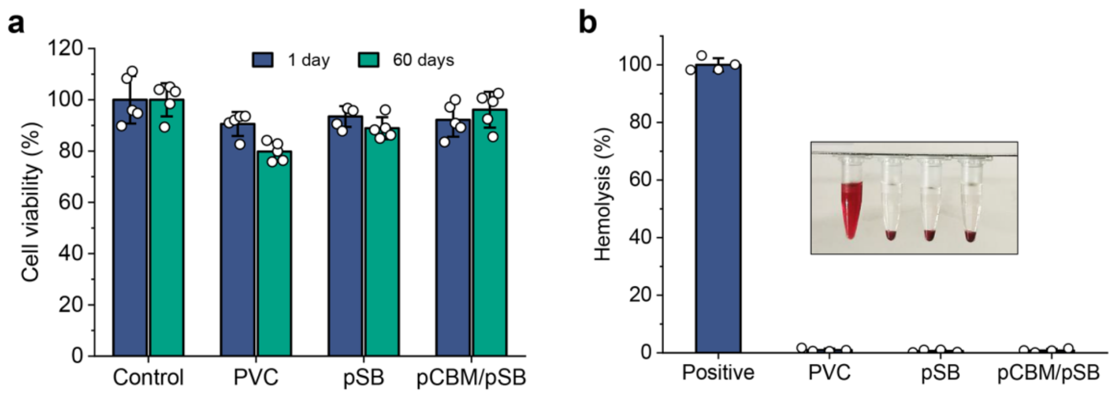

Supplementary Fig. 17. (**a**) Cytotoxicity (n=5) and (**b**) hemolysis rate (n=4) of PVC substrate, pSB hydrogel coating, and pCBM/pSB hydrogel coating. Data presented as mean ± SD in **a**-**b**. Source data are provided as a Source Data file.

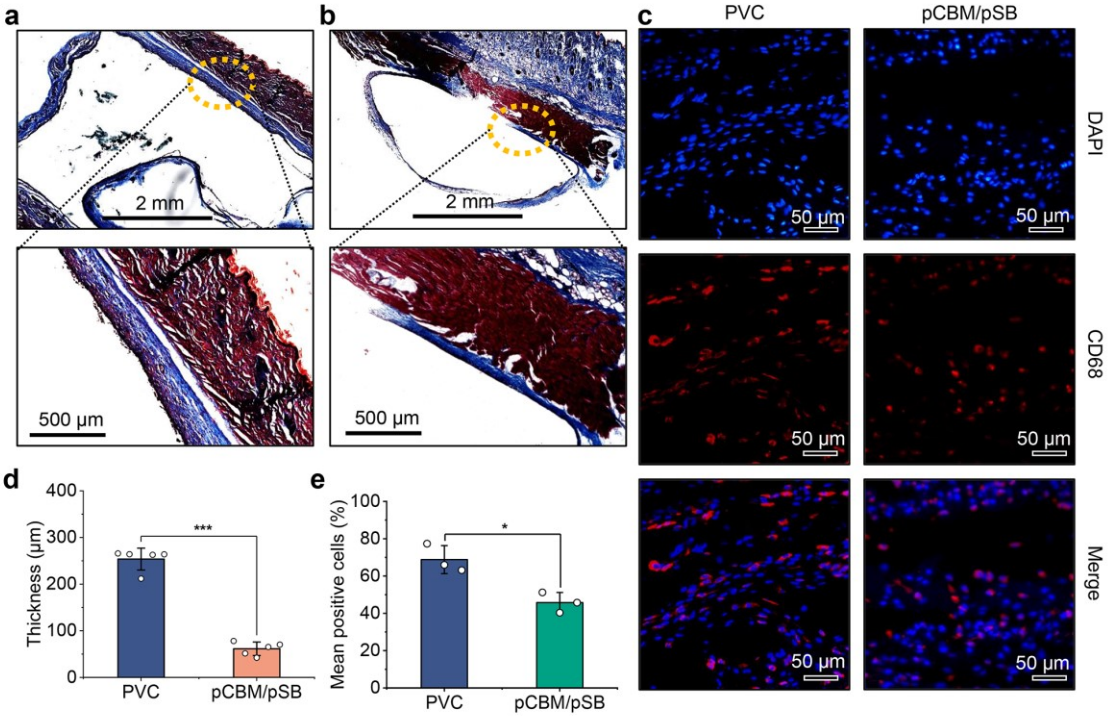

Supplementary Fig. 18. Representative Masson's trichrome staining images of (**a**) PVC and (**b**) pCBM/pSB hydrogel coatings after subcutaneous 30 d implantation. (**c**) CD68 (red) and DAPI (blue) staining for macrophages on 30 d post-operation. Measurements in **a-c** were repeated three times independently with similar results. (**d**) Fibrosis layer thickness (n=5) and (**e**) mean positive macrophages (n=3) of PVC and pCBM/pSB hydrogel coating. Data presented as mean ± SD and analyzed using a one-way ANOVA with Tukey's post hoc test in **d-e**, *$p < 0.05$, ***$p < 0.001$. **d** $p < 0.001$ (PVC vs pCBM/pSB, Thickness). **e** $p = 0.0126$ (PVC vs pCBM/pSB, Mean positive cells). Source data are provided as a Source Data file.

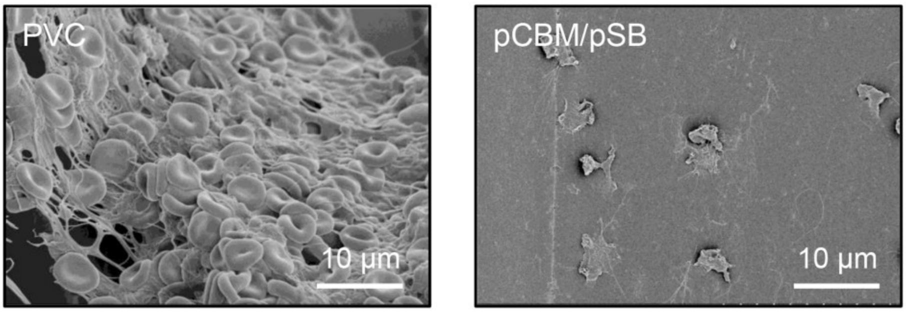

Supplementary Fig. 19. SEM images of pristine PVC tubing and PVC tubing coated with pCBM/pSB hydrogel coating after an *ex vivo* perfusion experiment in SD rats for 1 h. These measurements were repeated three times independently with similar results.

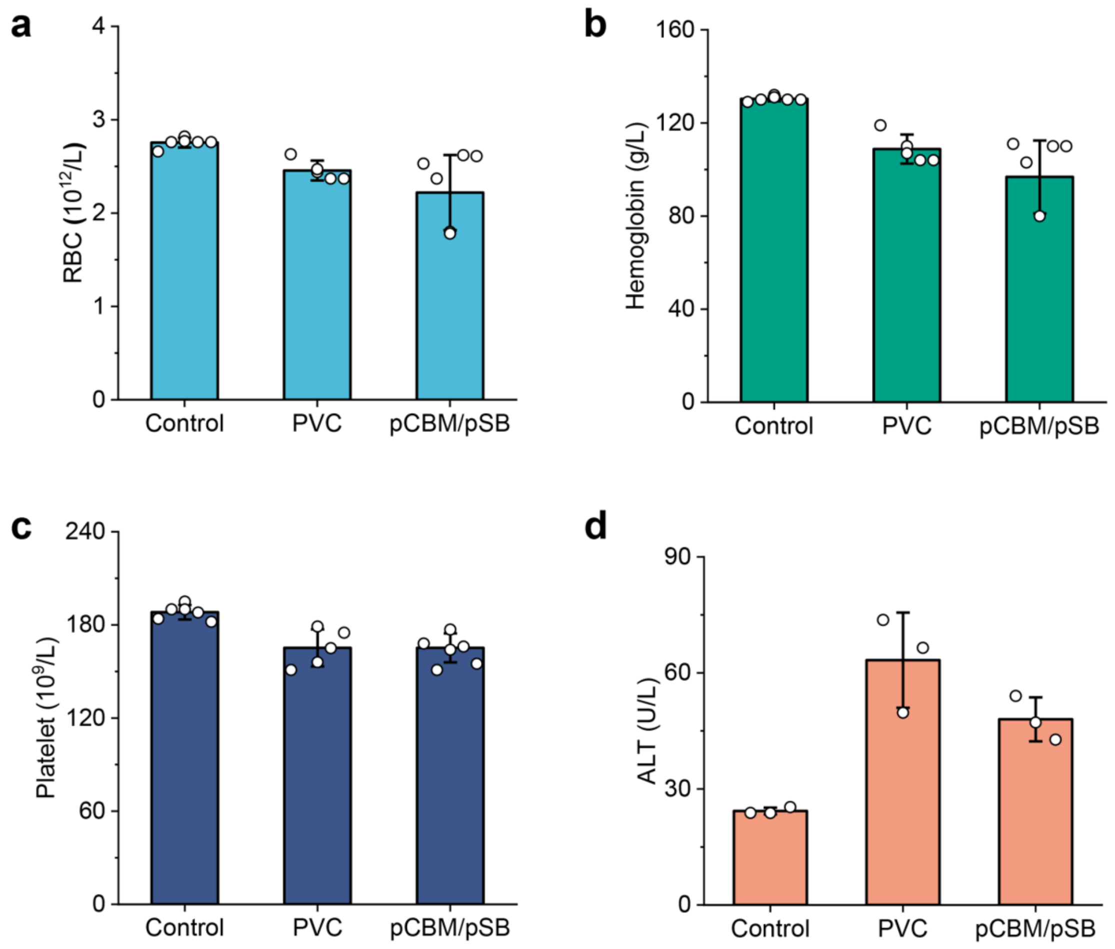

Supplementary Fig. 20. (**a**) The values of red blood cell (RBC) (n=5), (**b**) hemoglobin (n=5), (**c**) platelet (n=6), and (**d**) alanine aminotransferase (ALT) (n=3) of pristine PVC and pCBM/pSB hydrogel coating after an *ex vivo* perfusion experiment in a rabbit for 2 h. Data presented as mean ± SD in **a**-**d**. Source data are provided as a Source Data file.
