# Supplementary material for: Microgel reinforced zwitterionic hydrogel coating for blood-contacting biomedical devices
Source: Nat Commun. 2022 Sep 12;13:5339. doi: 10.1038/s41467-022-33081-7 (PMC9468150; doi:10.1038/s41467-022-33081-7)
Supplement: Supplementary file 2 — Reporting Summary [file 41467_2022_33081_MOESM2_ESM.pdf]

nature portfolio

Corresponding author(s): Junjie Li

Last updated by author(s): Aug 22, 2022

Reporting Summary

Nature Portfolio wishes to improve the reproducibility of the work that we publish. This form provides structure for consistency and transparency in reporting. For further information on Nature Portfolio policies, see our Editorial Policies and the Editorial Policy Checklist.

Statistics

For all statistical analyses, confirm that the following items are present in the figure legend, table legend, main text, or Methods section.

n/a	Confirmed	
☐	☒	The exact sample size (n) for each experimental group/condition, given as a discrete number and unit of measurement
☐	☒	A statement on whether measurements were taken from distinct samples or whether the same sample was measured repeatedly
☐	☒	The statistical test(s) used AND whether they are one- or two-sided<br>*Only common tests should be described solely by name; describe more complex techniques in the Methods section.*
☒	☐	A description of all covariates tested
☒	☐	A description of any assumptions or corrections, such as tests of normality and adjustment for multiple comparisons
☐	☒	A full description of the statistical parameters including central tendency (e.g. means) or other basic estimates (e.g. regression coefficient) AND variation (e.g. standard deviation) or associated estimates of uncertainty (e.g. confidence intervals)
☐	☒	For null hypothesis testing, the test statistic (e.g. F, t, r) with confidence intervals, effect sizes, degrees of freedom and P value noted<br>*Give P values as exact values whenever suitable.*
☒	☐	For Bayesian analysis, information on the choice of priors and Markov chain Monte Carlo settings
☒	☐	For hierarchical and complex designs, identification of the appropriate level for tests and full reporting of outcomes
☒	☐	Estimates of effect sizes (e.g. Cohen's d, Pearson's r), indicating how they were calculated

Our web collection on statistics for biologists contains articles on many of the points above.

Software and code

Policy information about availability of computer code

Data collection	Electromechanical Tester (WDW-05, Si Pai Inc, China), NIS-Elements Viewers (v4.2.2), Image J software (v1.6.0), Zeiss ZEN software (ver 3.4) was used for capture of immunofluorescence images
Data analysis	Origin 2021 software and Microsoft Excel 2016 were used for data plotting and some of the statistical analysis. Image J software were used for quantitative imaging analyses. Zeiss ZEN software (ver 3.4) was used for analysis of immunofluorescent images.

For manuscripts utilizing custom algorithms or software that are central to the research but not yet described in published literature, software must be made available to editors and reviewers. We strongly encourage code deposition in a community repository (e.g. GitHub). See the Nature Portfolio guidelines for submitting code & software for further information.

Data

Policy information about availability of data

All manuscripts must include a data availability statement. This statement should provide the following information, where applicable:

- Accession codes, unique identifiers, or web links for publicly available datasets
- A description of any restrictions on data availability
- For clinical datasets or third party data, please ensure that the statement adheres to our policy

The datasets generated during and/or analysed during the current study are available from the corresponding author on reasonable request.

Human research participants

Policy information about studies involving human research participants and Sex and Gender in Research.

Reporting on sex and gender	N/A
Population characteristics	N/A
Recruitment	N/A
Ethics oversight	N/A

Note that full information on the approval of the study protocol must also be provided in the manuscript.

Field-specific reporting

Please select the one below that is the best fit for your research. If you are not sure, read the appropriate sections before making your selection.

- [x] Life sciences
- [ ] Behavioural & social sciences
- [ ] Ecological, evolutionary & environmental sciences

For a reference copy of the document with all sections, see nature.com/documents/nr-reporting-summary-flat.pdf

Life sciences study design

All studies must disclose on these points even when the disclosure is negative.

Sample size	Each finding was confirmed with minimum necessary number such as 1-6 replicates for each experiments. The sample was randomly chooseto be determined. These sample sizes are sufficient which have reached statistical significance.
Data exclusions	No data was excluded in this study.
Replication	All experiments were performed with independent replicates as described in the figure legends.
Randomization	All samples were randomly allocated into experimental groups
Blinding	Investigators were blinded to group allocation during data collection and analysis.

Reporting for specific materials, systems and methods

We require information from authors about some types of materials, experimental systems and methods used in many studies. Here, indicate whether each material, system or method listed is relevant to your study. If you are not sure if a list item applies to your research, read the appropriate section before selecting a response.

Materials & experimental systems

n/a	Involved in the study	
[ ]	[x]	Antibodies
[ ]	[x]	Eukaryotic cell lines
[x]	[ ]	Palaeontology and archaeology
[ ]	[x]	Animals and other organisms
[x]	[ ]	Clinical data
[x]	[ ]	Dual use research of concern

Methods

n/a	Involved in the study	
[x]	[ ]	ChIP-seq
[x]	[ ]	Flow cytometry
[x]	[ ]	MRI-based neuroimaging

Antibodies

Antibodies used	Primary antibodies: Rabbit anti-rat CD68 (Abcam; catalog no. AB125212). Species Reactivity: Mouse,Rat; Application:IF;Secondary antibodies: Cy3–conjugated Affinipure Goat Anti-Rabbit IgG (H+L) (Proteintech, Cat. No.: SA00009-2) Species Reactivity: Mouse,Rat; Application:IF;
Validation	There is no novel antibodies in this study. All antibodies used were well described in literature or in the manufacturer'sprotocols. The protocols for antibody dilution and incubation time in the immunofluorescence assay were optimized.

Eukaryotic cell lines

Policy information about cell lines and Sex and Gender in Research

Cell line source(s)	L929 cell lines were purchased from iCell Bioscience (Shanghai, China).
Authentication	L929 cell lines weren't authenticated by the investigators in this paper. They were purchased from the company.
Mycoplasma contamination	L929 cell lines were not tested for mycoplasma contamination.
Commonly misidentified lines (See ICLAC register)	No commonly misidentified cell lines were used in the study

Animals and other research organisms

Policy information about studies involving animals; ARRIVE guidelines recommended for reporting animal research, and Sex and Gender in Research

Laboratory animals	Male Sprague-Dawley rats (SD rats, 8 weeks old) and Male New zealand white rabbits (~4 months old, 2.5-3.0 kg) were used for all our experiments.
Wild animals	This study did not involve wild animals.
Reporting on sex	All male animals were used in this study, and gender differences were not considered.
Field-collected samples	This study did not involve samples collected from the field.
Ethics oversight	The animal experiments were performed in accordance with the Guidelines for Care and Use of Laboratory Animals of the Tianjin Medical University General Hospital and approved by the Animal Ethics Committee of the Tianjin Medical University General Hospital (Tianjin, China).

Note that full information on the approval of the study protocol must also be provided in the manuscript.
